# Enhancing isoprenol production by systematically tuning metabolic pathways using CRISPR interference in *E. coli*

**DOI:** 10.3389/fbioe.2023.1296132

**Published:** 2023-11-06

**Authors:** Jinho Kim, Taek Soon Lee

**Affiliations:** ^1^ Joint BioEnergy Institute, Emeryville, CA, United States; ^2^ Biological Systems and Engineering Division, Lawrence Berkeley National Laboratory, Berkeley, CA, United States

**Keywords:** CRISPR interference, isoprenol, multiplexed CRISPRi arrays, mevalonate pathway, IPP-bypass pathway, fed-batch cultivation

## Abstract

Regulation of metabolic gene expression is crucial for maximizing bioproduction titers. Recent engineering tools including CRISPR/Cas9, CRISPR interference (CRISPRi), and CRISPR activation (CRISPRa) have enabled effective knock-out, knock-down, and overexpression of endogenous pathway genes, respectively, for advanced strain engineering. CRISPRi in particular has emerged as a powerful tool for gene repression through the use of a deactivated Cas9 (dCas9) protein and target guide RNA (gRNA). By constructing gRNA arrays, CRISPRi has the capacity for multiplexed gene downregulation across multiple orthogonal pathways for enhanced bioproduction titers. In this study, we harnessed CRISPRi to downregulate 32 essential and non-essential genes in *E. coli* strains heterologously expressing either the original mevalonate pathway or isopentenyl diphosphate (IPP) bypass pathway for isoprenol biosynthesis. Isoprenol remains a candidate bioproduct both as a drop-in blend additive and as a precursor for the high-performance sustainable aviation fuel, 1,4-dimethylcyclooctane (DMCO). Of the 32 gRNAs targeting genes associated with isoprenol biosynthesis, a subset was found to vastly improve product titers. Construction of a multiplexed gRNA library based on single guide RNA (sgRNA) performance enabled simultaneous gene repression, yielding a 3 to 4.5-fold increase in isoprenol titer (1.82 ± 0.19 g/L) on M9-MOPS minimal medium. We then scaled the best performing CRISPRi strain to 2-L fed-batch cultivation and demonstrated translatable titer improvements, ultimately obtaining 12.4 ± 1.3 g/L isoprenol. Our strategy further establishes CRISPRi as a powerful tool for tuning metabolic flux in production hosts and that titer improvements are readily scalable with potential for applications in industrial bioproduction.

## 1 Introduction

Microbial host engineering is a promising strategy for improving advanced biofuel production and increasing sustainability in the energy sector. Advanced biofuels are petrochemical analogues typically derived by microbial hosts grown on non-food based feedstocks (e.g., lignin, waste cooking oil, or syngas) with comparably low lifecycle greenhouse gas emissions (Keasling et al., 2021). To date, microbial ethanol remains one of a handful of first-generation biofuels to achieve commercialization owing in part to its metabolic simplicity as a byproduct of anaerobic fermentation. However, ethanol has significant physicochemical drawbacks relative to petroleum fuels, such that biofuel research has shifted towards more energy-dense molecules, including fatty acid methyl esters, higher alcohols, polyketides, and terpenes (Baral et al., 2021; Keasling et al., 2021).

Improving microbial titer, rate, and yield of more favorable and often more complex biofuels on recalcitrant carbon sources remains an outstanding challenge in metabolic engineering owing largely to the complexities of metabolic networks. A primary goal of metabolic engineering is to reroute metabolic flux towards a desired pathway while reducing inhibitory flux imbalances (Kim et al., 2017). Traditionally, *Eschericha coli* has been engineered by knockout of endogenous genes associated with competing byproducts or pathways via λ-Red recombineering (Datsenko and Wanner, 2000; Lee and Kim, 2015). However, consecutive gene knockouts are typically irreversible, strictly limited to non-essential genes, static, and laborious to multiplex. A more recent strategy for parallel gene editing is multiplex automated genome engineering (MAGE), which enables simultaneous modification of multiple genomic locations, including mismatches, insertions, and deletions (Wang et al., 2009). However, MAGE demands that hosts are deficient in DNA mismatch-repair and the frequency of variants harboring multiple phenotype improving mutations is often much lower than that of single-mutation variants (Wang et al., 2009; Gallagher et al., 2014). Furthermore, these strategies are often complicated by the unintuitive and nonobvious interplay between genetic expression owing to regulatory elements, enzyme promiscuity, substrate toxicity, and their collective impacts on targeted production.

The recent discovery of clustered regularly interspaced short palindromic repeats (CRISPR)-associated protein (Cas) has rapidly advanced precise RNA-guided genome engineering. Unlike previous genome engineering tools such as zinc finger nucleases (ZFNs) and transcription activator-like effector nucleases (TALENs), CRISPR/Cas9 systems enable efficient deletion, insertion, or modification at a target locus (Cho et al., 2018). Modifications of the canonical CRISPR/Cas9 system have enabled trans-acting gene modulation, namely, CRISPR activation (CRISPRa) and CRISPR interference (CRISPRi) systems for effective upregulation and downregulation of target genes for advanced strain engineering, respectively (Chae et al., 2017; Tan and Prather, 2017). In particular, CRISPRi utilizes a deactivated Cas9 (dCas9) protein and a guide RNA (gRNA) to downregulate transcription of a target gene without knockout (Wu et al., 2015; Kim et al., 2016; Tian et al., 2019). Consequently, CRISPRi systems can reversibly and dynamically modulate expression of both non-essential and essential genes to elucidate their relative impacts on a given production pathway. Downregulation of selected genes using CRISPRi has contributed to significant bioproduct titer improvements, including dyes (Kim et al., 2016; Czajka et al., 2022; Cho et al., 2023), flavonoids (Tao et al., 2018), nutraceuticals (Wu et al., 2017), and biofuels (Tian et al., 2019; Wang et al., 2022).

One such biofuel, isoprenol (3-methyl-3-buten-1-ol), has arisen as a promising renewable intermediate for high-volume bioblendstocks (Foo et al., 2014). Isoprenol has attractive physicochemical properties including higher energy content, lower water miscibility, hygroscopicity, and volatility compared to ethanol. Isoprenol can be acetylated to generate isoprenyl acetate, an octane boosting fuel blend additive (Carruthers et al., 2023). Furthermore, isoprenol can be easily converted to isoprene (by catalytic dehydration). Isoprene is a well-known precursor of synthetic rubber and recently it is identified as a precursor for the high-performance jet fuel blend additive 1,4-dimethylcyclooctane (DMCO) (Rosenkoetter et al., 2019; Baral et al., 2021).

Isoprenol production in *Escherichia coli* may be accomplished via either the native methylerythritol 4-phosphate (MEP) pathway or heterologous mevalonate (MVA) pathway. Although metabolically distinct, both pathways ultimately generate isopentenyl diphosphate (IPP), a universal precursor to isoprenoid biosynthesis. Sequential dephosphorylation of IPP to isoprenol is accomplished by the promiscuous activity of one or more endogenous *E. coli* phosphatases (George et al., 2015). Many subsequent strategies have attempted to eliminate metabolic bottlenecks by tuning phosphatase and MVA pathway expression to improve isoprenol biosynthesis (George et al., 2015). However, IPP accumulation by the heterologous MVA pathway remained a significant bottleneck due to its inhibition of cell growth and deleterious impact on isoprenol production (George et al., 2018). To address IPP toxicity, a phosphomevalonate decarboxylase (PMD) enzyme with promiscuous activity towards mevalonate monophosphate (MVAP) was adapted to bypass the formation of IPP, thereby avoiding toxic accumulation, reducing pathway ATP consumption, and ultimately enhancing overall isoprenol titers by 2.4-fold compared to wild-type (Kang et al., 2017; 2019). Further optimization of this “IPP-bypass” pathway by expression of gene variants achieved titers of 3.7 g/L and 10.8 g/L isoprenol under batch and fed-batch conditions at scale, respectively, which is the highest titers reported so far (Kang et al., 2019; Tian et al., 2019).

CRISPRi has been successfully used to modulate transcription of genes in the original MVA isoprenol biosynthesis pathway to improve titers by 98% (Tian et al., 2019) and, more recently, to downregulate *E. coli* genes in pathways competing for isoprenol precursors (Wang et al., 2022). While these studies improved isoprenol titer, they used rich medium, which makes it difficult for industrial scale-up, reshapes the cellular metabolic landscape dramatically, and often yields disparate sets of beneficial CRISPRi target genes.

**FIGURE 1:**
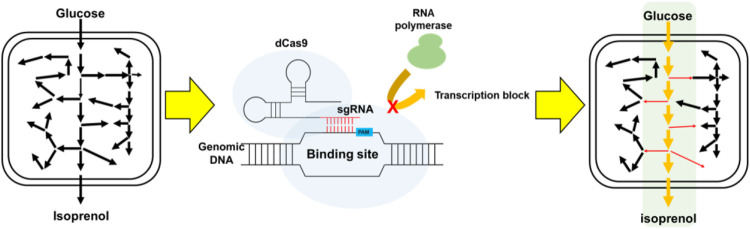
Schematic for the improvement of isoprenol biosynthesis by CRISPRi-mediated downregulation of competing pathways.

In this study, we harnessed CRISPRi methods to downregulate an expanded pool of endogenous genomic *E. coli* genes to improve isoprenol production titers in minimal medium via both the original MVA pathway and the IPP-bypass pathway. We then constructed multiplexed gRNA arrays leveraged for enhanced isoprenol biosynthesis with the best combinations yielding a 3 to 4.5-fold improvement in isoprenol via the IPP-bypass pathway. Finally, we show that the CRISPRi platform and the resultant titer improvements were scalable to 2-L bioreactors under fed-batch conditions, ultimately achieving 12.4 ± 1.3 g/L isoprenol in minimal medium (Figure 1). Broadly, our isoprenol titer improvements further establish CRISPRi as a powerful tool for microbial metabolic engineering.

## 2 Materials and methods

### 2.1 Plasmids and strains

The pdCas9-Marraffini plasmid (JBEI-18706) served as a template for our CRISPRi system (Bikard et al., 2013; Tian et al., 2019). The plasmid encodes a catalytically inactive dCas9 from *Streptococcus pyogenes* driven by an anhydrotetracycline (aTc) inducible promoter (P_tet_) along with a nontargetting spacer (5′-TGA​GAC​CAG​TCT​CGG​AAG​CTC​AAA​GGT​CTC-3′). The spacer is flanked by BsaI cut sites for integration of gRNAs via golden gate assembly (NEBridge Golden Gate Assembly Kit, New England Biolabs, United Kingdom). Target sgRNAs were designed using Benchling (http://benchling.com) and screening for PAM sites that were 1) on the non-template DNA strand, 2) proximal to the start codon or promoter region, and 3) unlikely to cause off-target effects. Selected 30 bp sgRNAs were integrated under the constitutive promoter pJ23119 (Tian et al., 2019). Multiplexed gRNA arrays were then constructed with gRNAs flanked by repeat sequences (5′-GTT​TTA​GAG​CTA​TGC​TGT​TTT​GAA​TGG​TCC​CAA​AAC-3′).

*E. coli* DH1 was selected as a production strain owing to demonstrated high titer production of isoprenol via either the original MVA pathway (George et al., 2018) or the IPP-bypass pathway (Kang et al., 2019).

All plasmids and strains used in this study are listed in Table 1 with gRNA nucleotide sequences listed in Supplementary Table S1. The plasmids and strains were deposited and are publicly available in the JBEI Registry (http://public-registry.jbei.org). All results in this study were also deposited into the Experiment Data Depot (EDD, http://edd.jbei.org).

**TABLE 1:** List of base strains and plasmids used in this study.

Strains	Description	Reference
*Isoprenol pathway strains*		
*E. coli* DH1	F^−^ supE44 hsdR17 recA1 gyrA96 relA1 endA1 thi^−1^ lambda^-^	Wild type
OriMVA	*E. coli* DH1 harboring JBEI-6829 and JPUB_004507	George et al. (2018)
IPP-By	*E. coli* DH1 harboring JBEI-9310 and JBEI-14559	Kang et al. (2019)

Plasmids	Description	Reference
*Isoprenol pathway plasmids*		
OriMVA-Upper	pLacUV5-atoB-HMGS_Sa-HMGR_Sa-MKco-PMKco, p15a, CmR	George et al. (2018)
OriMVA-Lower	pTrc99A-NudBR10-PMD_Sc, pBR322, AmR	George et al. (2018)
IPPBy-Upper	pLacUV5-atoB-HMGS_Sa-HMGR_Sa, p15a, CmR	Kang et al. (2019)
IPPBy-Lower	pTrc-PMD_R74H-R147K-M212Q, pBR322, AmR	Kang et al. (2019)
*Single gRNA plasmids* (pSC101 origin, KanR, dCas9 under pTet promoter, gRNA under pJ23119)		
Non-target Control (JBEI-18706)	pTet-dCas9-pJ23119-NT_Control, pSC101, KanR	Tian et al. (2019)
*accA*	pTet-dCas9-pJ23119-accA, pSC101, KanR	Tian et al. (2019)
*ackA*	pTet-dCas9-pJ23119-ackA, pSC101, KanR	Tian et al. (2019)
*adhE*	pTet-dCas9-pJ23119-adhE, pSC101, KanR	This study
*arcC*	pTet-dCas9-pJ23119-arcC, pSC101, KanR	Tian et al. (2019)
*asnA*	pTet-dCas9-pJ23119-asnA, pSC101, KanR	Tian et al. (2019)
*citE*	pTet-dCas9-pJ23119-citE, pSC101, KanR	This study
*dacA*	pTet-dCas9-pJ23119-dacA, pSC101, KanR	Tian et al. (2019)
*deoB*	pTet-dCas9-pJ23119-deoB, pSC101, KanR	Tian et al. (2019)
*eutD*	pTet-dCas9-pJ23119-eutD, pSC101, KanR	Tian et al. (2019)
*fabH*	pTet-dCas9-pJ23119-fabH, pSC101, KanR	This study
*gldA*	pTet-dCas9-pJ23119-gldA, pSC101, KanR	Tian et al. (2019)
*ispA*	pTet-dCas9-pJ23119-ispA, pSC101, KanR	Tian et al. (2019)
*ldhA*	pTet-dCas9-pJ23119-ldhA, pSC101, KanR	This study
*mdh*	pTet-dCas9-pJ23119-mdh, pSC101, KanR	Tian et al. (2019)
*menA*	pTet-dCas9-pJ23119-menA, pSC101, KanR	This study
*mqo*	pTet-dCas9-pJ23119-mqo, pSC101, KanR	Tian et al. (2019)
*pgl*	pTet-dCas9-pJ23119-pgl, pSC101, KanR	This study
*poxB*	pTet-dCas9-pJ23119-poxB, pSC101, KanR	This study
*ppc*	pTet-dCas9-pJ23119-ppc, pSC101, KanR	Tian et al. (2019)
*ppsA*	pTet-dCas9-pJ23119-ppsA, pSC101, KanR	This study
*prpE*	pTet-dCas9-pJ23119-prpE, pSC101, KanR	Tian et al. (2019)
*pta*	pTet-dCas9-pJ23119-pta, pSC101, KanR	Tian et al. (2019)
*rssA*	pTet-dCas9-pJ23119-rssA, pSC101, KanR	This study
*sdhABCD*	pTet-dCas9-pJ23119-sdhABCD, pSC101, KanR	This study
*sucA*	pTet-dCas9-pJ23119-sucA, pSC101, KanR	This study
*sucB*	pTet-dCas9-pJ23119-sucB, pSC101, KanR	This study
*sucCD*	pTet-dCas9-pJ23119-sucCD, pSC101, KanR	This study
*thrC*	pTet-dCas9-pJ23119-thrC, pSC101, KanR	Tian et al. (2019)
*ubiA*	pTet-dCas9-pJ23119-ubiA, pSC101, KanR	This study
*yahl*	pTet-dCas9-pJ23119-yahl, pSC101, KanR	Tian et al. (2019)
*yhfW*	pTet-dCas9-pJ23119-yhfW, pSC101, KanR	Tian et al. (2019)
*yqeA*	pTet-dCas9-pJ23119-yqeA, pSC101, KanR	Tian et al. (2019)
*Double gRNA plasmids* (pSC101 origin, KanR, dCas9 under pTet promoter, gRNA’s under pJ23119)		
*fabH-ubiA*	pTet-dCas9-pJ23119-fabH-ubiA, pSC101, KanR	This study
*adhE-menA*	pTet-dCas9-pJ23119-adhE-menA, pSC101, KanR	This study
*ldlhA-ubiA*	pTet-dCas9-pJ23119-ldhA-ubiA, pSC101, KanR	This study
*ldhA-fabH*	pTet-dCas9-pJ23119-ldhA-fabH, pSC101, KanR	This study
*ldhA-menA*	pTet-dCas9-pJ23119-ldhA-menA, pSC101, KanR	This study
*adhE-fabH*	pTet-dCas9-pJ23119-adhE-fabH, pSC101, KanR	This study
*ldhA-adhE*	pTet-dCas9-pJ23119-ldhA-adhE, pSC101, KanR	This study
*adhE-ubiA*	pTet-dCas9-pJ23119-adhE-ubiA, pSC101, KanR	This study
*fabH-menA*	pTet-dCas9-pJ23119-fabH-menA, pSC101, KanR	This study
*menA-ubiA*	pTet-dCas9-pJ23119-menA-ubiA, pSC101, KanR	This study
*ppsA-adhE*	pTet-dCas9-pJ23119-ppsA-adhE, pSC101, KanR	This study
*ppsA-fabH*	pTet-dCas9-pJ23119-ppsA-fabH, pSC101, KanR	This study
*ppsA-ldhA*	pTet-dCas9-pJ23119-ppsA-ldhA, pSC101, KanR	This study
*ppsA-menA*	pTet-dCas9-pJ23119-ppsA-menA, pSC101, KanR	This study
*ppsA-ubiA*	pTet-dCas9-pJ23119-ppsA-ubiA, pSC101, KanR	This study
*Triple gRNA plasmids*		
*adhE-fabH-ldhA*	pTet-dCas9-pJ23119-adhE-fabH-ldhA, pSC101, KanR	This study
*adhE-fabH-menA*	pTet-dCas9-pJ23119-adhE-fabH-ldhA, pSC101, KanR	This study
*adhE-fabH-ubiA*	pTet-dCas9-pJ23119-adhE-fabH-ldhA, pSC101, KanR	This study
*menA-ubiA-fabH*	pTet-dCas9-pJ23119-menA-ubiA-fabH, pSC101, KanR	This study

### 2.2 Batch production of isoprenol

All batch and fed-batch isoprenol production experiments were completed in M9-MOPS defined medium Briefly, M9-MOPS minimal medium contains M9 salts (33.9 g/L Na_2_HPO_4_, 15 g/L KH_2_PO_4_, 5 g/L NH_4_Cl, and 2.5 g/L NaCl; Sigma-Aldrich) supplemented with 75 mM MOPS, 2 mM MgSO_4_, 1 mg/L thiamine, 10 nM FeSO_4_, 0.1 mM CaCl_2_, and micronutrients (Korz et al., 1995) including 3*10^−8^ M (NH_4_)_6_Mo_7_O_24_, 4*10^−6^ M boric acid, 3*10^−7^ M CoCl_2_, 1*10^−7^ M CuSO_4_, 8*10^−7^ M MnCl_2_, and 1*10^−7^ M ZnSO_4_ with 20 g/L of glucose as the sole carbon source. Three antibiotics (100 mg/L carbenicillin, 30 mg/L chloramphenicol, and 100 mg/L kanamycin) were used for selection where necessary (Kang et al., 2019; Tian et al., 2019).

*E. coli* DH1 was transformed with either the original MVA or IPP-bypass pathway plasmids as well as a dCas9-gRNA plasmid via electroporation (2500 V, 5 ms) in 2 mm gap cuvettes (Bio Rad), recovered at 37°C for 1 hour in SOC medium, then plated on antibiotic for overnight outgrowth. A successful transformant was inoculated into Luria-Bertani (LB) liquid medium with antibiotic at 37°C overnight for each transformation. When preparing the glycerol stock, we inoculated a significant tiny single colony from the agar plate into LB culture medium, then the cell was cultured to ensure they did not exceed OD_600_ of 2 in the LB medium. Cultures were passaged 50-fold (v/v) in fresh M9-MOPS medium, grown overnight, and passaged again into fresh M9-MOPS medium to ensure adaptation from complex to minimal medium. Adapted cells were stored as frozen glycerol cryo stocks at −80°C. The OD_600_ of glycerol stock is approximately 2, and we uniformly conducted all experiments by inoculating this strain into fresh M9-MOPS minimal medium with an initial OD_600_ of 0.2 to minimize the genetic mutations.

Cells were recovered from glycerol cryo stocks as needed by direct inoculation into glass culture tubes containing 5 mL M9-MOPS medium supplemented with 20 g/L glucose and grown at 37°C overnight. Overnight cultures were diluted 50-fold (v/v) into 5 mL fresh M9-MOPS medium and grown for 12 h at 37°C. Finally, cells were inoculated at an optical density (OD; λ = 600 nm, 1 cm path length) of 0.2 into 5 mL fresh M9-MOPS medium. When culture OD_600_ was between 0.6 and 0.8, isoprenol biosynthesis and dCas9 expression were simultaneously induced by addition of 0.5 mM isopropyl β-D-1-thiogalactopyranoside (IPTG) and 10 nM anhydrotetracycline (aTc), respectively. Induced cultures were then grown at 30°C for 72 h. Samples were taken at 48 h and 72 h to measure strain growth rates using OD_600_ (SpectraMax 384, Molecular Devices) and isoprenol titer (Focus GC-FID, Thermo Scientific).

### 2.3 Isoprenol production in fed-batch cultivation

As in batch experiments, cells from a frozen glycerol stock were inoculated into 2 mL M9-MOPS medium and grown for 12 h at 37°C, then diluted 50-fold into 5 mL fresh M9-MOPS medium for overnight growth. The seed culture was then passed into 100 mL M9-MOPS medium, which was grown for 8 h at 37°C and transferred to 900 mL fresh M9-MOPS medium in a 2-L bioreactor vessel at an OD_600_ of 0.3.

Fed-batch cultivation was conducted using a 2-L bioreactor (Sartorius BIOSTAT B plus) with control of dissolved oxygen (DO), temperature, and pH. DO and airflow were set to 30% and 1 VVM (volume of air per volume of liquid per minute), respectively, with temperature maintained at 30°C and pH held at 6.5 by supplementation with 25% ammonia water. The CRISPRi system was induced by addition of 10 nM aTc and isoprenol biosynthesis was induced by addition of 0.5 mM IPTG as in batch culture. For fed-batch mode, a mixture of 80 g/L glucose and 15 g/L ammonium chloride was supplied using a Watson-Marlow DU520 peristaltic pump. After the lag phase, the feeding flow rate was calculated following Korz’s equation and increased every hour for a total of 6 h as described by Equation 1 (Korz et al., 1995; Kang et al., 2019).

$$m_{s}\left(t\right)=\left(\frac{\mu }{Y_{\frac{x}{s}}}+m\right)V_{t_{F}}*X_{t_{F}}*e^{\mu *\left(t-t_{F}\right)}$$ (1)

Here, $m_{s}\left(t\right)$ is the flow of glucose (g/hr) and μ represents the specific growth rate (hr^-1^) of the *E. coli* strain. V_tF_ is the cultivation volume (L) and X_tF_ is biomass (g/L) at feeding time t_F_ (hr), while Y_x/s_ is the yield of biomass on substrate (g_biomass_/g_glucose_) and m is the specific maintenance coefficient (g_biomass_/g_glucose_/hr). Glucose concentration was measured consistently using a glucose meter (CVSHealth) and high-performance liquid chromatography (HPLC) during exponential feeding. Following exponential feeding, a feed rate was selected that closely matched glucose consumption such that the glucose concentration was less than 1 g/L.

A 20% oleyl alcohol overlay was directly added to the vessel at the time of induction, serving both to extract isoprenol and to mitigate isoprenol evaporation due to bioreactor sparging. Antifoam B was added during the fed-batch phase as necessary.

### 2.4 Quantification of isoprenol

Isoprenol was quantified from both the aqueous culture medium as well as the organic oleyl alcohol overlay. For aqueous extraction, a 250 μL aliquot of cell culture was vigorously mixed with 250 μL of ethyl acetate containing 1-butanol (30 mg/L) internal standard by vortexing at 3000 RPM for 15 min. After vortexing, the mixture was centrifuged at 14,000 x g for 2 min to separate organic and aqueous phases. A 100 μL aliquot of the ethyl acetate was then diluted 5-fold into fresh ethyl acetate with internal standard for analysis using gas chromatography with flame ionization detection (GC-FID; Focus GC-FID, Thermo Scientific) with 1 μL injection volume into a DB-WAX column (15 m, 0.32 mm inner diameter, 0.25 μm film thickness, Agilent). The GC-FID oven temperature was initially set to 40°C, then ramped to 100 °C at a rate of 15 °C/min, ramped to 230°C at a rate of 40°C/min, and finally held at 230°C for 3 min.

Organic phase isoprenol concentration was determined by sampling 250 μL of the oleyl alcohol overlay and centrifuging at 14,000 x g for 5 min to separate any aqueous cell culture. Then 10 μL of the overlay was added to 990 μL ethyl acetate with an internal standard for GC-FID analysis. Isoprenol titers under fed-batch conditions with overlay were calculated on the basis of actual culture volume at the time of the sampling.

### 2.5 Quantification of glucose, acetate, and ethanol

High performance liquid chromatography (HPLC; Agilent Technologies) was performed to quantify glucose, ethanol, and acetate in aqueous cultures. Aqueous culture samples were centrifuged at 14,000 x g for 5 min to separate supernatant and cells. Following centrifugation, 100 μL of supernatant was filtered (0.45 μm centrifugal filter) and analyzed using an HPLC system equipped with an Aminex HPX-87H column (Bio-rad) and a refractive index detector. The sample tray temperature was set to 10°C and the column oven temperature was set to 65°C with a 0.005 M H_2_SO_4_ mobile phase flow rate of 600 μL/min. Data acquisition and analysis were performed via Agilent Chemstation.

## 3 Results

### 3.1 Preparing isoprenol production pathways

**FIGURE 2:**
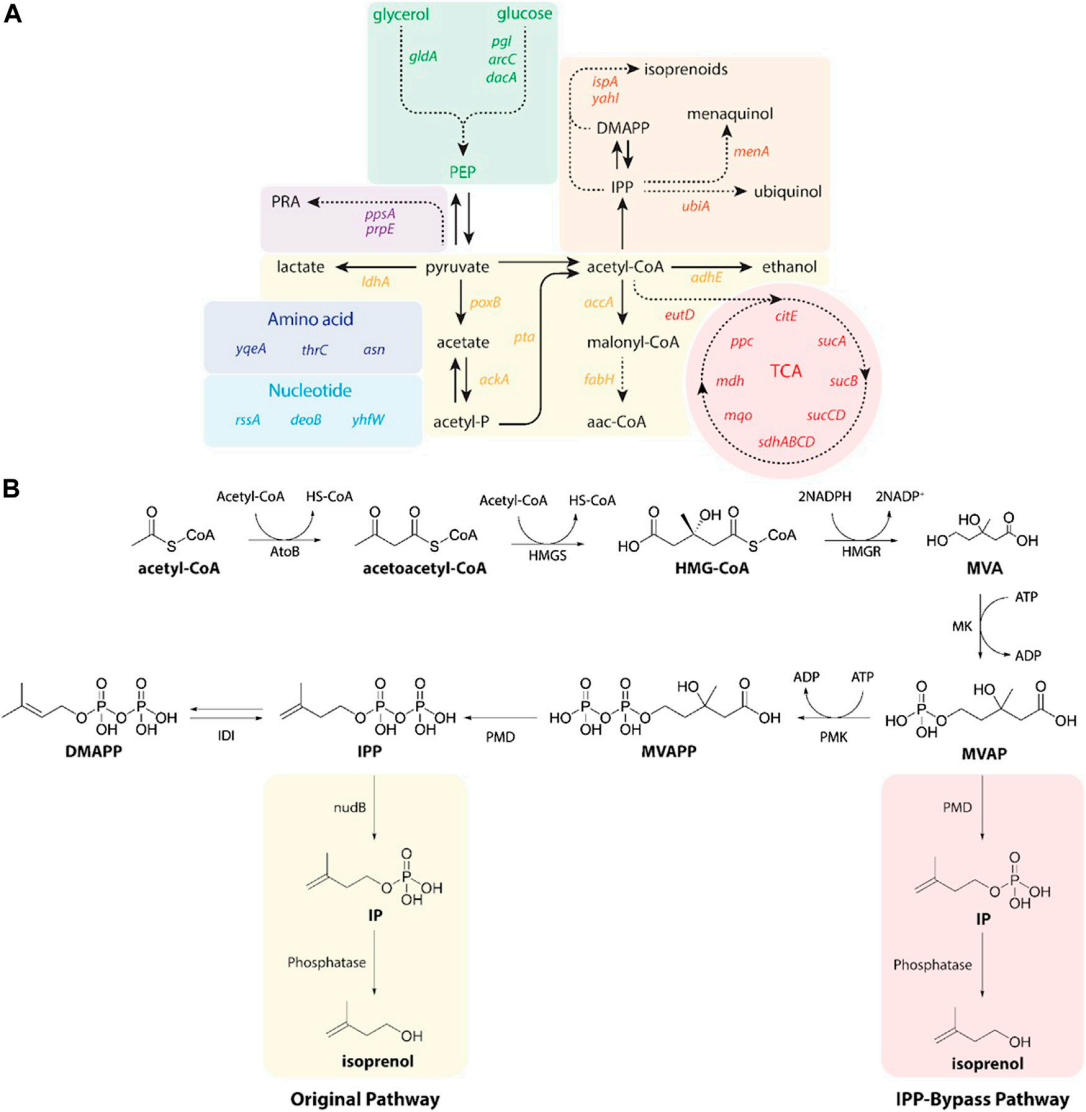
**(A)** A pathway map of gene knockdown targets for redirecting metabolic flux toward isoprenol production using CRISPRi. Pathways and relevant genes are colored as follows: green box, glycolysis; purple box, amino acids pathway; violet box, propionic acid pathway; light blue box, nucleotides pathway; yellow box, organic acids pathway; orange box, isoprenoids pathway; red box, TCA cycle pathway. PRA, propionic acid; PEP, phosphoenolpyruvate; TCA, citric acid cycle. **(B)** Original (yellow box) and IPP-bypass (red box) MVA pathways for isoprenol production. The MVA pathway converts acetyl-CoA to IPP in 6 enzymatic steps with subsequent isomerization to dimethylallyl diphosphate (DMAPP). Dephosphorylation of IPP and DMAPP by nudB, a promiscuous *E. coli* phosphatase, produces isoprenol and prenol, respectively. The IPP-bypass pathway was proposed in this study for direct decarboxylation of mevalonate monophosphate (MVAP) to isopentenyl monophosphate (IP) followed by dephosphorylation by an endogenous phosphatase. ADP, adenosine diphosphate; AtoB, acetoacetyl-CoA thiolase; ATP, adenosine triphosphate; DMAPP, dimethylallyl diphosphate; HMG-CoA, 3-hydroxy-3-methyl-glutaryl-CoA; HMGR, HMG-CoA reductase; HMGS, HMG-CoA synthase; IDI, isopentenyl diphosphate isomerase; IP, isopentenyl monophosphateIPP, isopentenyl diphosphate; MK, mevalonate kinase; MVA, mevalonate; MVAP, mevalonate monophosphate; MVAPP, mevalonate diphosphate; PMD, mevalonate diphosphate kinase; PMK, phosphomevalonate kinase.

Isoprenol was produced by strains harboring either the original MVA pathway (George et al., 2015) or the IPP-bypass MVA pathway (Kang et al., 2019; 2017) as shown in Figure 2B. In brief, both pathway variants share 4 genes (*atoB*, *HMGS*, *HMGR*, and *MK*) which convert the central metabolite acetyl-CoA to mevalonate phosphate (MVAP) (Supplementary Figure S2). The original MVA pathway forms isopentenyl diphosphate (IPP) from MVAP by mevalonate phosphate kinase (PMK) and phosphomevalonate decarboxylase (PMD), and the IPP is hydrolyzed to isoprenol sequentially by native promiscuous phosphatases (NudB and other monophosphatase).

The accumulation of IPP is toxic to the production host, however, (George et al., 2018), and the IPP-bypass pathway was designed to produce isoprenol using promiscuous activity of PMD without forming IPP (Kang et al., 2016). In the IPP-bypass pathway strain, the expression of 4 genes of the MVA pathway (*atoB*, *HMGS*, *HMGR*, and *MK*) was controlled under the P_lacUV5_ on the plasmid 1 and the evolved PMD mutant (triple mutants, R74H-R147K-M212Q) (Kang et al., 2017) was overexpressed under P_trc_ on the plasmid 2 (Supplementary Figure S1).

### 3.2 Designing CRISPRi system and sgRNA library of target genes

In this study, we used the CRISPRi strategy to identify genes associated with improved isoprenol production by attenuating gene expression of the competing metabolic pathways. The dCas9-sgRNA system is a simple and effective method for partial or completed gene downregulation by blocking transcription. The SC101 origin and tetracycline-inducible promoter were employed for high efficiency stability of the plasmid and dCas9 expression (Tian et al., 2019). The use of the inducible promoter system has allowed for the selective inhibition of genes, even for those essential genes, and this work has also demonstrated that the effects of targeted gene suppression last for an extended period (at least up to 72 h). We designed to induce the heterologous isoprenol biosynthesis pathway concurrently with the CRISPRi induction, so that endogenous target genes in competing pathways are downregulated in parallel with the pathway overexpression for the isoprenol production.

To maximize the efficiency of the CRISPRi system, we designed the sgRNAs to target non-template DNA strands of the specific target gene after the protospacer adjacent motif (PAM) sequence (5′-NGG-3′) at the open reading frame (ORF) or promoter region as previously reported (Tian et al., 2019). We selected 32 genes as targets for downregulation as they competitively use precursors, cofactors or the intermediates of the MVA pathway (i.e., acetyl-CoA, pyruvate precursors, and cofactors) (Figure 2A). A subselection of these 32 candidate target genes have been downregulated by CRISPRi to improve isoprenol titer in a previous study using a complex or rich medium and only the original MVA pathway (Tian et al., 2019). First, we selected 15 gene targets that compete with isoprenol biosynthesis for acetyl-CoA and pyruvate precursors, including *accA* (acetyl-coenzyme A carboxyl transferase), *ackA* (acetate kinase), *adhE* (aldehyde alcohol dehydrogenase), *citE* (citrate lyase), *fabH* (3-oxoacyl-acyl-carrier protein synthase), *ldhA* (D-lactate dehydrogenase), *mdh* (malate dehydrogenase), *poxB* (pyruvate dehydrogenase), *ppc* (phosphoenolpyruvate carboxylase), *ppsA* (phosphoenolpyruvate synthase), *pta* (phosphate acetyltransferase), *sdhABCD* (succinate dehydrogenase), *sucA* (2-oxoglutarate dehydrogenase), *sucB* (2-oxoglutarate dehydrogenase), and *sucCD* (succinyl-CoA synthetase) (Wu et al., 2015; Kim et al., 2016; Tian et al., 2019). In addition, *asnA* (aspartate ammonia ligase), *gldA* (glycerol dehydrogenase), *pgl* (6-phosphogluconolactonase), and *prpE* (propionyl-CoA synthase) were also chosen as targets to enhance production of isoprenol as tested in the previous study (Tian et al., 2019). We added 3 genes, *ispA* (farnesyl diphosphate synthase), *menA* (1,4-dihydroxy-2-naphthoate octaprenyltransferase), and *ubiA* (4-hydroxybenzoate octaprenyltransferase) that compete for IPP utilization (Kong and Lee, 2011; Zada et al., 2018) and selected additional 10 genes including *araC* (arabinose regulator), *dacA* (alanine carboxypeptidase), *deoB* (deoxyribose), *eutD* (phosphotransacetylase), *mqo* (quinone-oxidoreductase), *rssA* (sigma S regulator protein), *thrC* (threonine synthase), *yahI* (polypeptide carbamate kinase), *yhfW* (mutase), and *yqeA* (carbamate kinase) that were previously identified as knockout targets in the lycopene and carotenoid production via the MEP pathway (Alper et al., 2005; Alper and Stephanopoulos, 2008; Tian et al., 2019). A detailed metabolic map of these genes in their cellular context is depicted in Supplementary Figure S2.

### 3.3 Knockdown of single target genes in *E. coli* harboring the MVA pathway

When applying the CRISPRi system to downregulate metabolic genes and enhance isoprenol production, the selection of an appropriate growth medium could be a pivotal determinant. Downregulating the expression of essential genes impedes the metabolic pathway. *E. coli*’s response to this, however, could be hampered by its nutrient-absorbing behavior if a complex rich medium is used for its growth (Liu et al., 2020). To minimize this effect, we used a minimal medium for all of our experiments.

**FIGURE 3:**
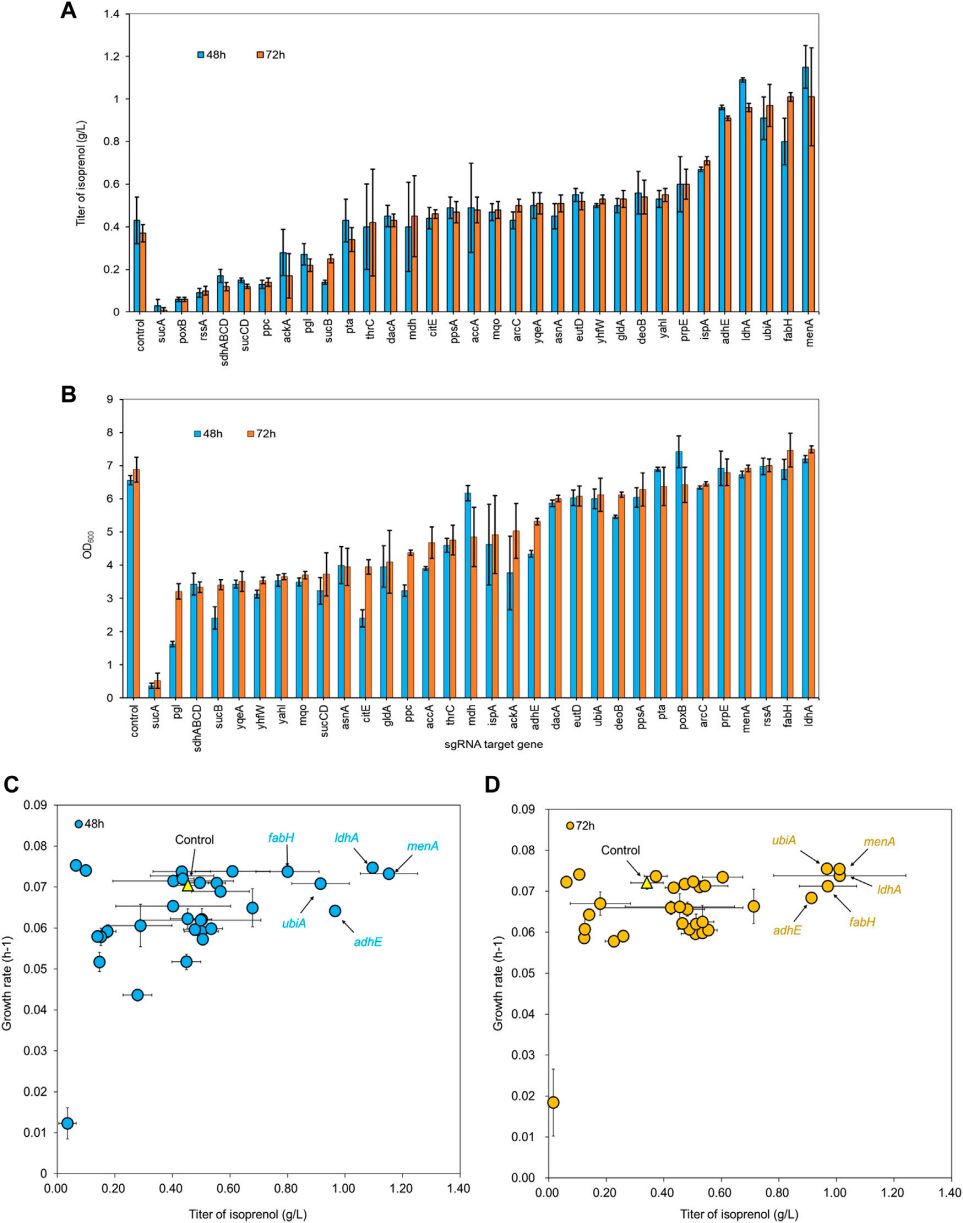
**(A)** Growth and **(B)** isoprenol production by strains harboring single gRNAs and the original MVA pathway. Batch cultures were grown in 5 mL M9-MOPS minimal medium supplemented with 20 g/L of glucose (n = 3). **(C)** Scatter plots of growth rate and isoprenol titer at 48 h and **(D)** 72 h show that selected gRNA targets were leveraged for high isoprenol production while maintaining a high growth rate relative to the control. The control harboring non-targeting gRNA plasmid JBEI-18706 is demarcated with a bright yellow triangle.

We transformed a library of 32 sgRNA-dCas9 plasmids into *E. coli* DH1 strains harboring the original MVA pathway to explore whether CRISPRi-mediated downregulation of selected target genes could improve isoprenol titer in minimal medium (Alper et al., 2005; Alper and Stephanopoulos, 2008; Kong and Lee, 2011; Wu et al., 2015; Kim et al., 2016; Zada et al., 2018; Tian et al., 2019). The growth rates of the strains were calculated to assess the relative burden of gene downregulation against a non-targeting control. Overall, the result showed that 22 out of 32 downregulation experiments for individual sgRNA targets resulted in higher isoprenol titers compared to the control strain harboring non-targeting gRNA plasmid JBEI-18706 (Figure 3A). Notably, sgRNAs targeting *adhE, ldhA, ubiA, fabH,* and *menA* yielded improved productions to 245.40%, 259.84%, 260.85%, 271.68%, and 271.94%, respectively, at isoprenol titers of 0.91 ± 0.01 g/L, 0.96 ± 0.02 g/L, 0.97 ± 0.10 g/L, 1.01 ± 0.02 g/L and 1.00 ± 0.23 g/L, respectively (Figure 3A).

Interestingly, the strains with the highest isoprenol titer harbor sgRNAs targeting orthogonal pathways: *ldhA* and *adhE* are involved in lactate and ethanol production, respectively, *fabH* is involved in fatty acid biosynthesis, and *ubiA* and *menA* utilize isoprenoid precursors for cellular respiration. Broadly speaking, these pathways are directly or indirectly involved in the consumption of the precursors used in the biosynthesis of isoprenol. For example, downregulation of *adhE, ldhA,* and *fabH* could reduce flux toward corresponding competing pathways and channel more precursor towards isoprenol biosynthesis. This is supported by the ethanol and lactate levels which were not detected in the culture medium when *adhE* and *ldhA* were downregulated while 0.8 ± 0.03 g/L of ethanol and 0.4 ± 0.03 g/L of lactate were detected in the control strain (Supplementary Figure S3).

Likewise, downregulation of *ldhA*, *adhE*, and *fabH* could also affect overall cellular metabolism and redox balance. These genes are involved in metabolic pathways that generate reducing equivalents (NADH or NADPH) or consume ATP, and the downregulation of these genes could result in altered metabolic fluxes, cellular redox state, and energy availability. It is important to note that unintended consequences of sgRNA-mediated gene downregulation could also be a contributing factor to the observed increase in isoprenol titer. Off-target effects or secondary effects on other cellular processes could indirectly impact isoprenol biosynthesis. Further studies, such as transcriptomic, proteomic, or metabolomic analyses, could provide a comprehensive understanding of the effects of *ldhA*, *adhE*, and *fabH* downregulation on cellular metabolism and isoprenol production.

In addition, *menA* and *ubiA* downregulation resulted in a significant increase in isoprenol titer. *MenA* is involved in the biosynthesis of menaquinone, an important cofactor in the electron transport chain, while *UbiA* is an essential enzyme in the ubiquinone biosynthesis pathway (Goodall et al., 2018). Both menaquinone and ubiquinone, which are vital components for cellular respiration and crucial to energy production, are derived from IPP, and downregulation of *menA* and *ubiA* could increase IPP availability towards isoprenol biosynthesis without compromising necessary function.

Conversely, sgRNAs targeting glycolysis pathway genes *rssA*, *ppc*, and *pgl* yielded a dramatic decrease in isoprenol production. In addition to decreasing isoprenol production, sgRNAs targeting TCA cycle genes *sucA*, *sucB*, *sucCD*, and *sdhABCD* also resulted in a significant growth inhibition (Figure 3B). This could be due to the repression of genes involved in sugar utilization, which may limit the availability of energy and precursor molecules for isoprenol biosynthesis. Alternatively, it's possible that these sgRNAs caused off-target effects or unintended consequences on cellular metabolism, leading to reduced isoprenol production, but more studies with extensive omics data will be needed to verify this.

In summary, the results suggest that sgRNAs targeting *adhE, ldhA*, *ubiA, fabH, and menA* provide a net benefit to isoprenol production via the MVA pathway and therefore may be valuable targets for further engineering efforts. On the other hand, those targeting TCA cycle and glycolysis pathway genes are strictly detrimental to the isoprenol titer improvement.

An overall comparison of the growth rates and isoprenol production at 48 h (Figure 3C) and 72 h (Figure 3D) across the 32 sgRNA experiments indicated these 5 genes with higher isoprenol titer and comparable growth rate to the control. We chose these five genes to serve as candidates for multiplexed gRNA arrays to elucidate potential synergistic effects to improve isoprenol titers.

### 3.4 Multiple gRNA arrays co-expression with the original MVA pathway

We created gRNA arrays by assembling five individual sgRNAs (*adhE, ldhA, ubiA, fabH, and menA*) into 10 double gRNA arrays (*adhE*/*ldhA*, *adhE*/*ubiA*, *adhE*/*fabH*, *adhE*/*menA*, *ldhA*/*ubiA*, *ldhA*/*fabH*, *ldhA*/*menA*, *ubiA*/*fabH*, *ubiA*/*menA*, and *fabH*/*menA*) to conduct a repression of multiple endogenous genes in *E. coli*.

**FIGURE 4:**
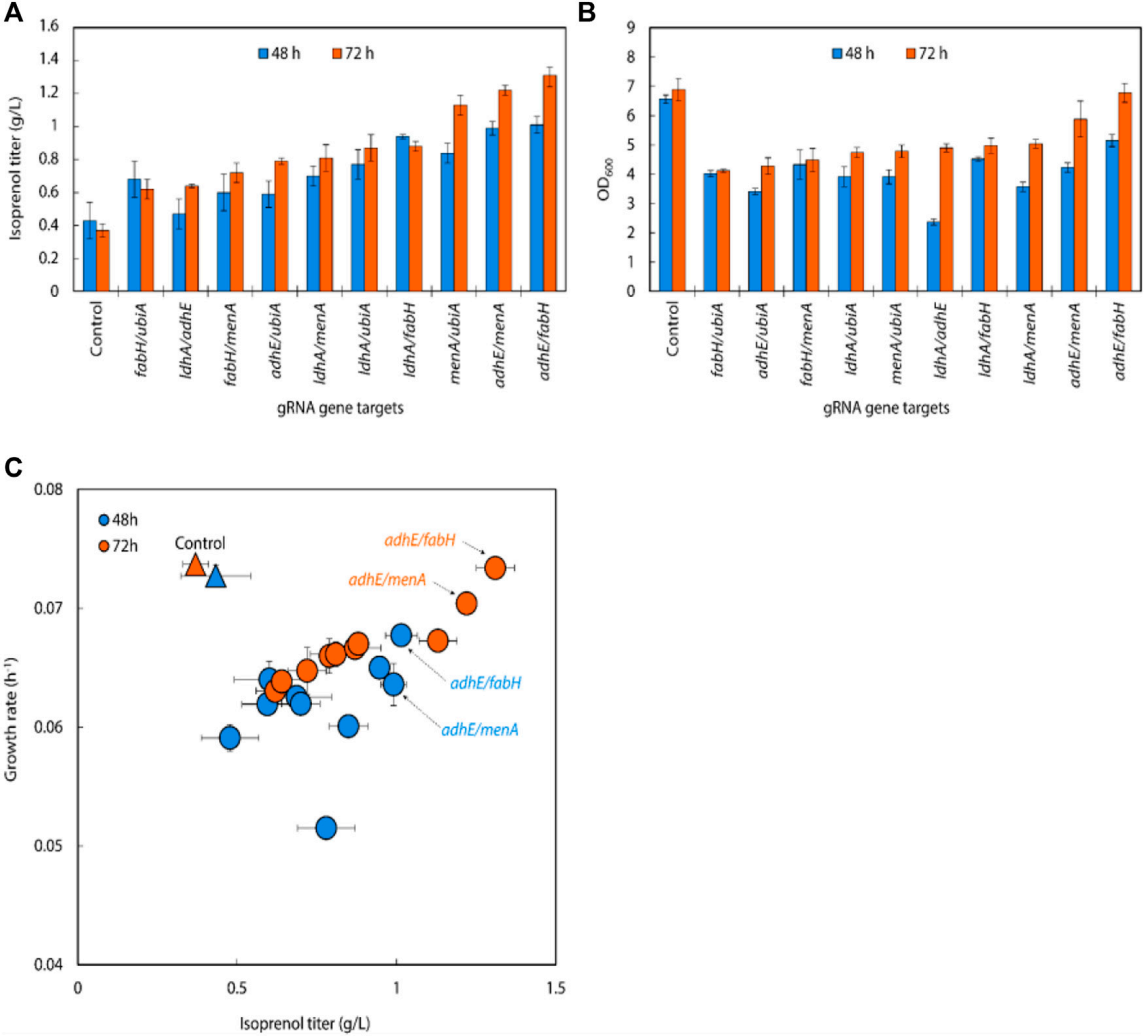
**(A)** Growth and **(B)** isoprenol production by strains harboring double gRNAs along with the original MVA pathway. Batch cultures were grown in 5 mL M9-MOPS minimal medium supplemented with 20 g/L of glucose (n = 3). **(C)** A scatter plot of growth rate and isoprenol titer at 48 h and 72 h and again show certain double gRNA strains significantly improved isoprenol production relative to the controls. The control at 48 h and 72 h is demarcated with appropriately colored triangles.

In the 10 double gRNA arrays we tested, three (*menA*/*ubiA*, *adhE*/*menA* and *adhE*/*fabH*) resulted in 20%–30% improvement of isoprenol titers over their respective single sgRNAs, attaining isoprenol titers of 1.13 ± 0.06 g/L, 1.22 ± 0.03 g/L and 1.31 ± 0.06 g/L, respectively (Figure 4A). This suggests that certain sgRNA combinations can synergistically redirect metabolic flux towards isoprenol production. Interestingly, we also observed that simultaneous downregulation of double gRNAs significantly reduced *E. coli* growth rate compared to the control strain (Figure 4B). This suggests that simultaneous gene repression poses a metabolic cellular burden, negatively affecting their overall fitness and growth. Further investigation is warranted to better understand the underlying mechanisms and optimize the sgRNA expression and regulation for improved isoprenol production without compromising cellular growth. In contrast, the strain targeting *adhE*/*fabH* exhibited similar growth rate compared to the control strain, indicating that the repression of *adhE*/*fabH* genes does not impose a significant metabolic burden on the cells. This may explain why the *adhE*/*fabH* strain showed comparable growth and isoprenol production levels, making it a promising candidate for further optimization and scale-up studies.

**FIGURE 5:**
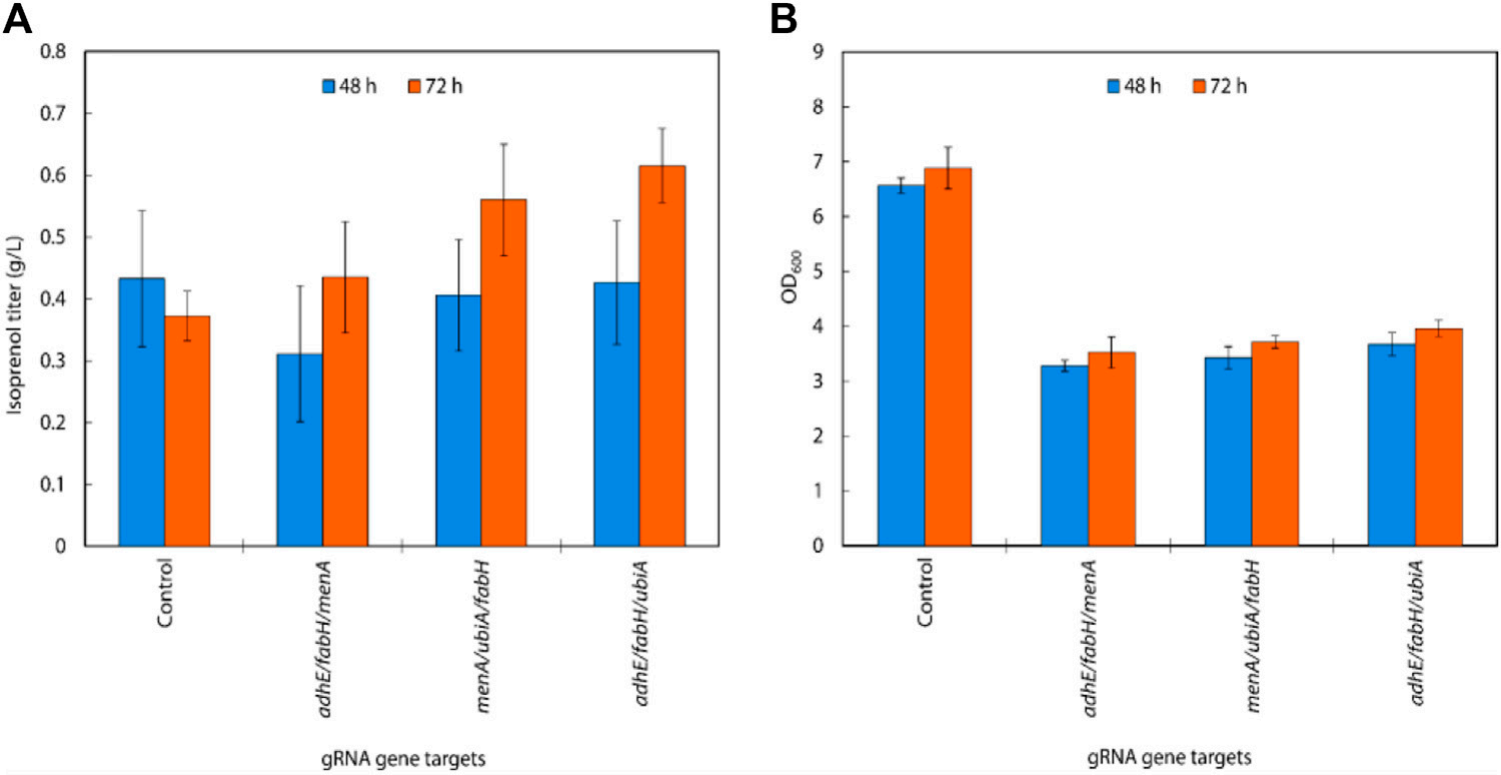
**(A)** Isoprenol production and **(B)** growth of strains harboring triple gRNAs along with the original MVA pathway grown in 5 mL M9-MOPS minimal medium supplemented with 20 g/L of glucose (n = 3).

Using the above results, we created gRNA arrays by assembling four individual sgRNAs (*adhE, ubiA, fabH, and menA*) into 4 triple gRNA arrays (*adhE/ubiA/fabH, adhE/ubiA/menA, adhE/ubiA/menA,* and *ubiA/fabH/menA*) to conduct multiple repression of endogenous genes in *E. coli* (Figure 4C). When the triple gRNAs were simultaneously downregulated, the growth rate of *E. coli* and the production of isoprenol were significantly reduced compared to those of double gRNAs strains even though they are still slightly higher than those of the control (Figure 5A). Interestingly, the strain with gRNA array targeting *adhE*/*menA*/*ubiA* did not grow (data not shown). Isoprenol titers of *adhE/fabH/menA, menA/ubiA/fabH, and adhE/fabH/ubiA* knock-down strains were 0.44 ± 0.09 g/L, 0.56 ± 0.09 g/L, and 0.62 ± 0.06 g/L, respectively (Figure 5A), with an approximately 50% reduction in growth compared to the control (Figure 5B).

The growth and isoprenol titer reduction of the triple gRNA strains relative to the control and the double gRNA strains that target the combination of the same set of genes (*adhE*, *fabH*, *menA*, and *ubiA*) suggests multiple gene downregulation in general is somewhat burdensome to cellular fitness. Further investigation could be conducted to elucidate the specific mechanism through which the simultaneous repression of multiple genes affects cellular fitness and isoprenol production.

### 3.5 Single gRNA library co-expression with the IPP-bypass pathway

**FIGURE 6:**
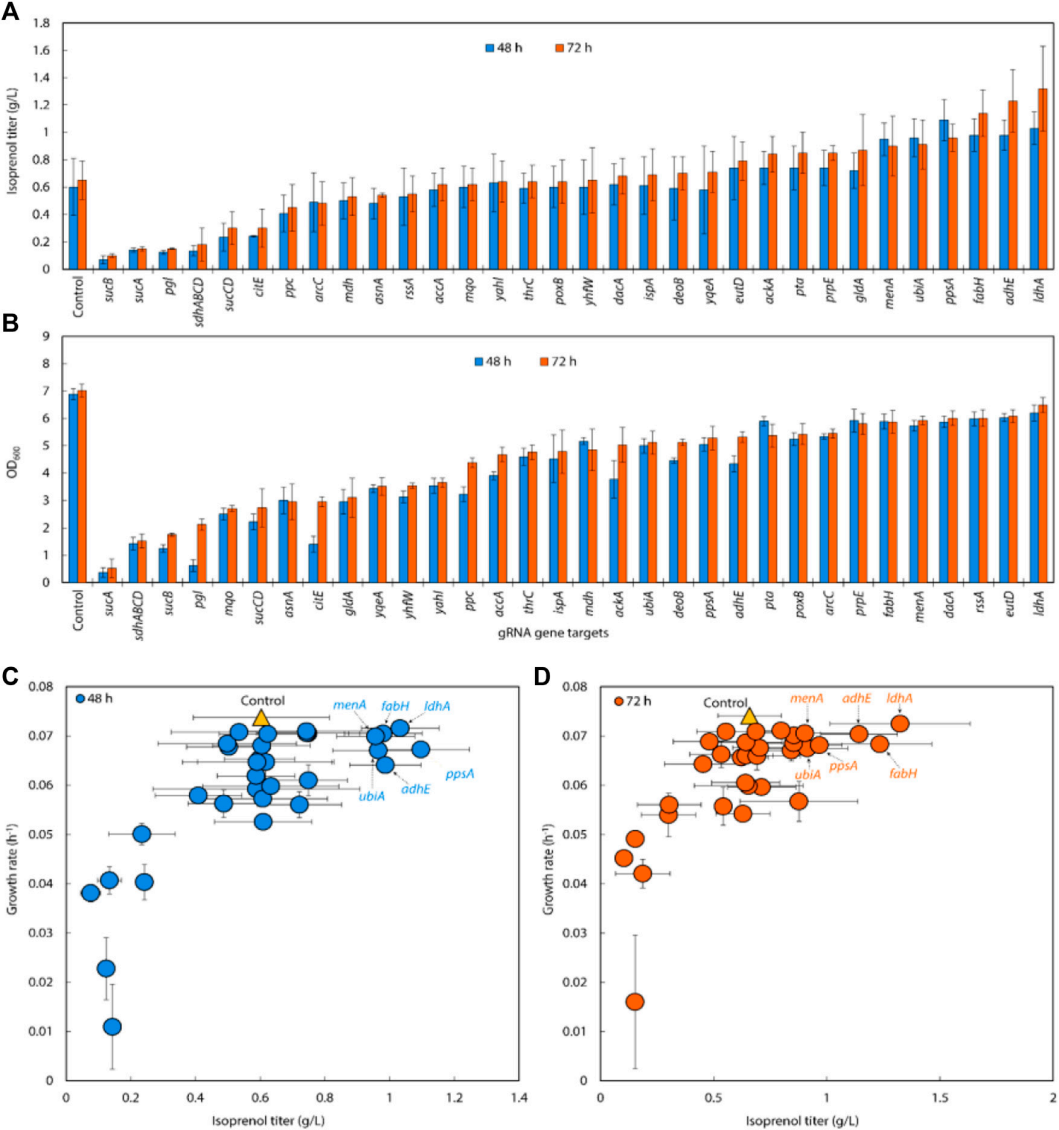
**(A)** Isoprenol production and **(B)** growth of strains harboring single gRNAs and the IPP-bypass pathway. Cultures were grown in 5 mL M9-MOPS minimal medium supplemented with 20 g/L of glucose (n = 3). **(C)** Scatter plots of growth rate and isoprenol titer at 48 h and **(D)** 72 h show that gene attenuation by certain gRNAs contributed to significant titer improvements, tracking well with observations from the original MVA pathway experiments. The control is demarcated with a yellow triangle.

Following the successful result of single, double, and triple gRNA arrays experiments in strains with the original MVA pathway, we transformed the same 32 sgRNAs library into *E. coli* DH1 strains with the IPP-bypass isoprenol production pathway and tested how the same gene knock down library behave in the slightly different isoprenol producing system. When co-expressing the IPP-bypass pathway and single gRNA library, 15 out of 32 sgRNAs harboring strains demonstrated higher isoprenol titers compared to the control with non-targeting gRNA (Figure 6A).

Similar to the results from the original MVA pathway, the IPP-bypass strains with sgRNAs targeting *menA*, *ubiA*, *ppsA, fabH,* and *adhE,* showed increases in isoprenol production by 136.93%, 138.52%, 146.62%, 173.24%, and 187.27%, with titers of 0.90 ± 0.22 g/L, 0.91 ± 0.18 g/L, 0.96 ± 0.10 g/L, 1.14 ± 0.17 g/L, and 1.23 ± 0.23 g/L, respectively (Figure 6A). The highest titer, however, was obtained from the strain with sgRNAs targeting *ldhA* with almost two-fold increased titer at 1.32 ± 0.31 g/L. Interestingly, this gene target did not show any titer improvement in the original MVA pathway experiment. We also found that the growth was similar to or slightly lower than that of the control strain when we downregulated *fabH* and *ldhA* expression using gRNAs (Figure 6B), while the growth of these strains was slightly higher than the control in the experiment with the original MVA pathway.

When downregulating glycolysis pathway and TCA cycle genes in the IPP-bypass pathway, we again observed growth inhibition and lower isoprenol titers confirming that genes on the glycolysis and the TCA cycle are not viable downregulation targets for isoprenol titer improvement. The reduction in growth of the strain upon downregulation of these genes implies that these pathways play a crucial role in the growth of *E. coli* and emphasizes the importance of maintaining the TCA cycle for survival before supplying the metabolic intermediates necessary for isoprenol production.

We generated a scatter plot of growth and isoprenol production of single gRNA arrays to construct the multiple gRNA arrays. Based on this analysis, the 6 genes (*adhE*, *ldhA*, *ppsA*, *ubiA*, *fabH*, and *menA*) were identified as a candidate group of sgRNAs with high isoprenol production and growth rate (Figures 6C,D).

### 3.6 Multiple gRNA arrays co-expression with the IPP-bypass pathway

**FIGURE 7:**
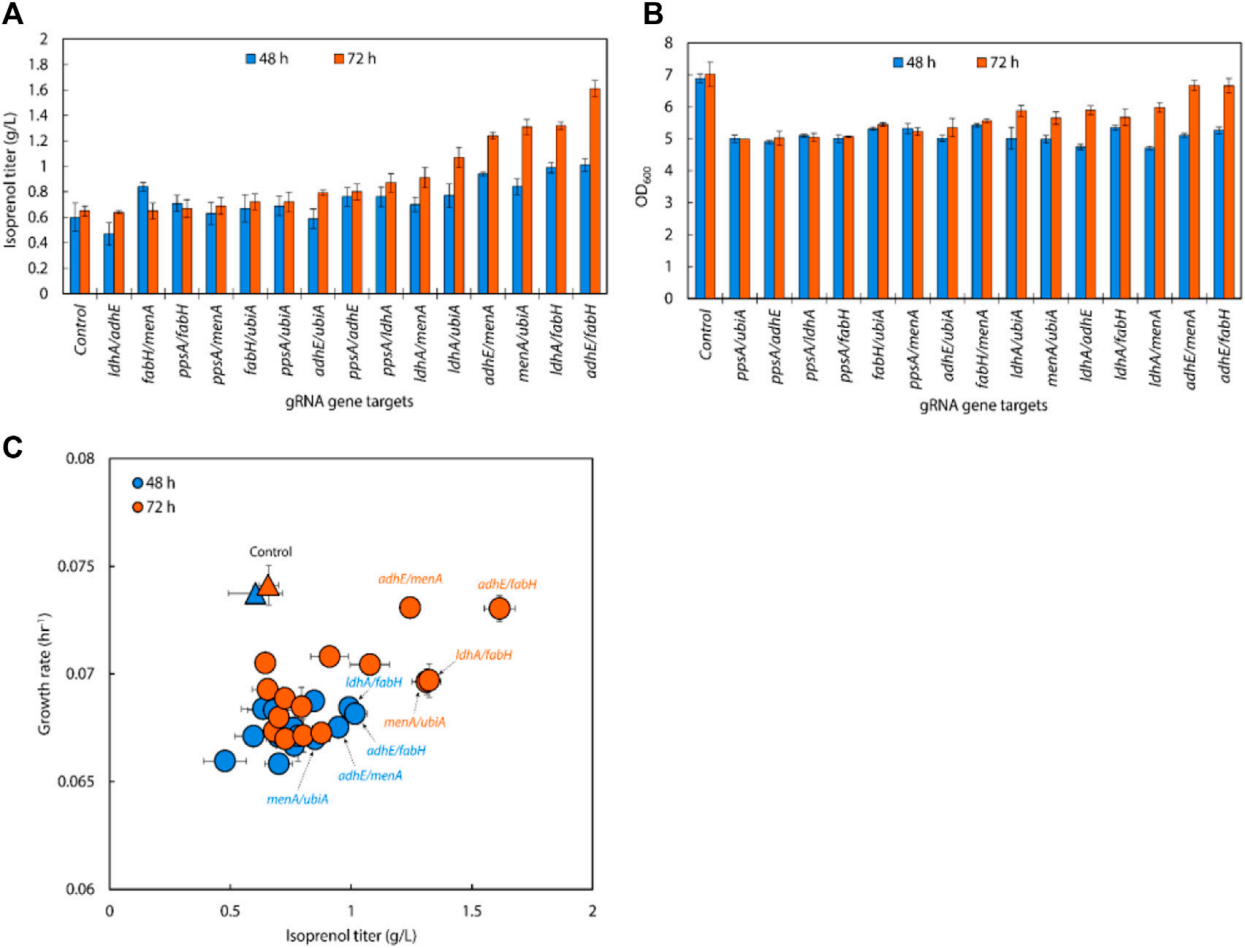
**(A)** Isoprenol production and **(B)** growth of strains harboring double gRNAs and the IPP-bypass pathway. Cultures were grown in 5 mL M9-MOPS minimal medium supplemented with 20 g/L of glucose (n = 3). **(C)** A scatter plot of growth rate and isoprenol titer at 48 h and 72 h show significant titer improvements by double gRNA harboring strains compared to the control. The control at 48 h and 72 h is demarcated with appropriately colored triangles.

Following the results of the single gRNA library on the IPP-bypass pathway strains, we assembled six individual gRNAs (*adhE, ldhA, ppsA, ubiA, fabH, and menA*) leveraged for higher isoprenol titer into all 15 possible double gRNA arrays (*adhE*/*ldhA*, *adhE*/*ppsA*, *adhE*/*ubiA*, *adhE*/*fabH*, *adhE*/*menA*, *ldhA*/*ppsA*, *ldhA*/*ubiA*, *ldhA*/*fabH*, *ldhA*/*menA*, *ppsA*/*ubiA*, *ppsA*/*fabH*, *ppsA*/*menA*, *ubiA*/*fabH*, *ubiA*/*menA*, and *fabH*/*menA*) to see whether multiplexed (i.e., double) endogenous gene repression could provide synergistic titer improvements in the IPP-bypass pathway strains. As shown in Figure 7A, the simultaneous targeting of *menA*/*ubiA*, *adhE*/*menA* and *adhE*/*fabH* resulted in significantly higher isoprenol titers than the control with non-targeting gRNA, achieving 1.31 ± 0.06 g/L, 1.32 ± 0.03 g/L and 1.61 ± 0.06 g/L, respectively. This production levels are higher than the previous production level increase by individual gene knockdown, and this result suggests that repression of *menA* and *ubiA*, *adhE* and either *menA* or *fabH* gene may more effectively redirect metabolic flux towards isoprenol production than when these genes are downregulated individually. The highest titer was achieved with a simultaneous downregulation of *adhE*/*fabH* and this titer improvement is attributed to synergistic effects of dual repression, resulting in a more pronounced enhancement of isoprenol production.

Interestingly, the results also show that downregulating the target genes of *adhE*/*fabH* and *adhE*/*menA* individually did not significantly affect the growth rate of *E. coli* compared to the control strain (Figure 7B). However, we observed a significant increase in the isoprenol titer when these genes were downregulated. These findings suggest that the *adhE* gene may not be essential for the growth of *E. coli* under the tested growth conditions. However, the *fabH* and *menA* are well known as essential for the growth of *E. coli* (Dhiman et al., 2019; Lai and Cronan, 2003; Beld et al., 2015; Rousset et al., 2021). This is consistent with previous research that has shown that these genes are involved in the production of fatty acid derivatives and menaquinone, respectively, which are important for cell membrane formation and energy production in bacteria. The significant increase in the isoprenol titer when these genes were downregulated suggests that *adhE*/*fabH* and *adhE*/*menA* may be competing for metabolic resources required for isoprenol production. By downregulating these genes individually, we may have reduced the metabolic burden on the cell, allowing more resources to be dedicated to isoprenol biosynthesis.

An analysis of double gRNA downregulation showed high isoprenol production by *adhE*/*fabH*. Based on comparison of growth rate and production titers (Figure 7C; Figure 6C), we then assembled three triple gRNA arrays that paired *adhE/fabH* with each of *ldhA*, *menA*, and *ubiA*.

**FIGURE 8:**
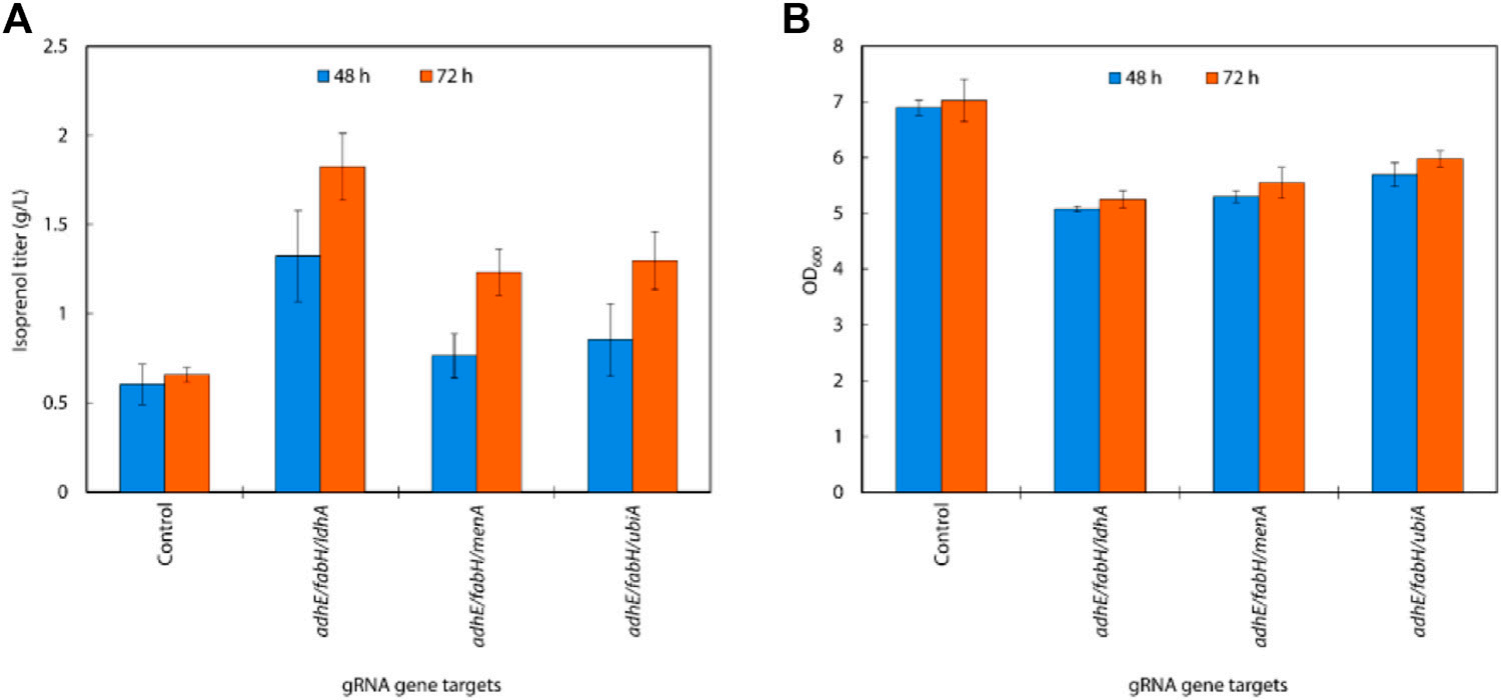
**(A)** Growth and **(B)** isoprenol production by strains harboring triple gRNAs and the IPP-bypass pathway grown in 5 mL M9-MOPS minimal medium supplemented with 20 g/L of glucose (n = 3).

Isoprenol production was significantly increased compared to the single and the double gRNAs strains with the constitutive sgRNAs with the best performing combination, *adhE*/*fabH*/*ldhA*, resulting in an isoprenol titer of 1.82 ± 0.19 g/L (Figures 8A,B). This juxtaposition suggests that acetyl-CoA accumulation is more crucial for enhancing isoprenol production in the IPP-bypass pathway than the accumulation of IPP.

As reported previously, the accumulation of IPP in the mevalonate pathway can be toxic to bacteria (George et al., 2018). By redirecting the flux of IPP away from the mevalonate pathway, *E. coli* was able to avoid the toxic effects of IPP accumulation and improved isoprenol production.

The increased production of isoprenol in the triple gRNA strains compared to the control strain and double gRNA strains indicates the potential of this multiplexed gRNAs approach for enhancing metabolic pathways in microbial cells.

### 3.7 Fed-batch cultivation of the multiple genes downregulated isoprenol strain

**FIGURE 9:**
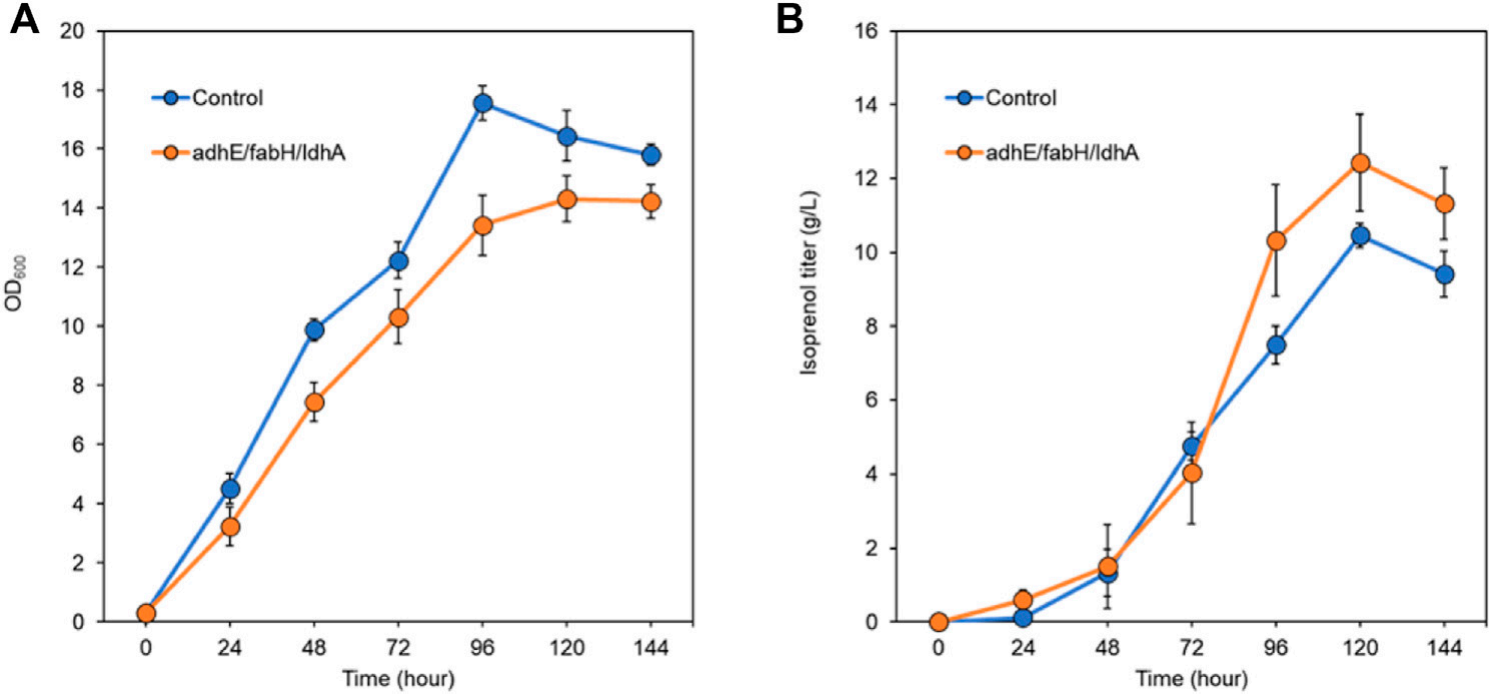
Isoprenol production and growth under fed-batch conditions by the control strain (harboring non-target gRNA plasmid (JBEI-18706)) and the triple gRNA strain targeting *adhA*, *fabH*, and *ldhA*. **(A)** growth of control and the strain harboring triple gRNA arrays. **(B)** production of isoprenol by the control strain and the strain harboring triple gRNA arrays. All strains include the IPP-bypass biosynthesis pathway plasmids. The fed-batch productions were performed in the 2-L bioreactors including M9-MOPS medium and 20% oleyl alcohol overlay at duplicate. Error bar represents standard deviation.

After screening the titer improvement by CRISPRi-mediated downregulation of genes in two isoprenol biosynthesis pathways, we selected the best performing strain, *E. coli* DH1 harboring the IPP-bypass pathway and triple gRNA targeting *adhE*, *ldhA*, and *fabH*, and scaled up the isoprenol production at a 2-L bioreactor under fed-batch conditions. Similar to what we observed in the 5 mL culture tube experiments, the strain harboring the CRISPRi system with the triple gRNAs array showed a slower growth rate and a higher maximum isoprenol production titer than the control strain harboring non-targeting gRNA plasmid JBEI-18706 (Figures 9A,B). In fed-batch cultivation of the control strain, the maximum cell growth was achieved at 96 h with an OD_600_ of 17.5 ± 0.5 and the maximum isoprenol production was achieved at 120 h with isoprenol titer of 10.4 ± 0.5 g/L (Figure 9A). The triple gRNAs array strain showed a slightly slower growth rate compared to the control strain, but about 20% increased isoprenol titer after 96 h with the highest titer of 12.4 ± 1.3 g/L and OD_600_ of 14.3 ± 0.7 at 120 h. The slower growth rate and the higher isoprenol production of the engineered strain in fed-batch condition confirms that the CRISPRi with *adhE*/*ldhA*/*fabH* gRNAs system worked and positively influenced the metabolic pathway for isoprenol biosynthesis not only at the small volume batch condition but also at the large scale fed-batch condition and led to improved production.

The use of fed-batch cultivation with continuous feeding of an additional glucose and ammonium chloride solution aimed to provide a sustained supply of carbon and nitrogen sources and contribute to the enhanced isoprenol production in the engineered strain. However, the rapid evaporation of isoprenol by agitation and air flow in the bioreactor remains a challenge in achieving higher titer. Even though our final titer was measured at 12.4 ± 1.3 g/L at 120 h, we observed a rapid decrease of the isoprenol level after 120 h and we also smelled a strong scent of isoprenol throughout the cultivation which suggests a significant evaporational loss from offgas of the bioreactor. Further optimization of the cultivation conditions such as optimization of the feeding strategy and the product recovery strategy both to improve the solvent extraction process and to capture isoprenol from the offgas could lead to higher isoprenol titers.

## 4 Discussion

In this work, we applied a combinatorial multiplex repression system using CRISPRi to downregulate endogenous genes in competing pathways for isoprenol production in *E. coli*. We demonstrated the success of the single and multiple CRISPRi system to improve isoprenol production in *E. coli*. The downregulation of target genes resulted in a considerable increase in isoprenol production, indicating that the CRISPRi system can modulate gene expression without adversely affecting cellular functions or knocking out essential genes. The observed increase in production may be attributed to reduced competition for precursors and altered cellular metabolism and redox balance. Furthermore, our results highlight the potential of using sgRNA arrays for multiple gene repression to modulate cellular metabolism and enhance isoprenol production in *E. coli*. The specific combinations of sgRNAs can significantly impact isoprenol titers, although careful consideration of their effects on cellular growth and fitness is necessary. This study also shows that the CRISPRi system is applicable to bench-top scale fermentation and the fed-batch condition with improvement of target bioproducts.

Interestingly, the identified downregulation gene targets that improved the isoprenol production were somewhat different from those in the previous report by Tian et al., 2019 which showcased the CRISPRi approach using an EZ-Rich defined medium and the report by Wang et al. which used an M9 medium but in conjunction with yeast extract (Wang et al., 2022). As previously reported by Goodall et al., disparities in culture media give rise to variations in gene expression (Goodall et al., 2018). This phenomenon likely explains the non-replication of genes such as *ackA*, *poxB*, and *pta* from Tian et al.’s study and genes *yggV* and *accA* from Wang et al.’s work. These differences underscore the potential impact of culture medium on genetic outcomes.

A nuanced constraint of CRISPRi-mediated downregulation lies in the inherent bias of the sgRNA library towards genes with anticipated functional relevance for titer enhancement, encompassing precursors, intermediates, and energy-related factors. However, comprehending precisely why gene downregulation amplifies the production beyond their initial functions remains a challenge. Consequently, meaningful analysis necessitates library-scale assessment of growth and production data rankings. To attain deeper insights, the synergy of CRISPRi, metabolomics, proteomics or transcriptomics, and machine learning becomes imperative. This fusion promises a more coherent rationale behind the beneficial effects of downregulation on production. Acquiring a finer grasp of altered metabolic networks could potentially enable time-dependent gene downregulation to refine biosynthesis.

In this study, we were able to identify the genes involved in improving isoprenol production by testing a limited number of single and multiple gRNAs. As knocking-out multiple genes is not a trivial task and gene knockouts frequently result in growth retardation, the gene knockdown strategy using CRISPRi is a powerful tool to screen multiple combinations of target genes. However, screening various combinations of multiple target genes is still not a trivial task as the total number of combinations easily reaches an enormous level. Therefore, it is quite important to build an automated process to generate a library of multiplex gRNA arrays and to perform accurate and reproducible experiments in a high throughput manner. The data generated by this automated platform will make predictions using artificial intelligence feasible and can significantly reduce experimental variables and accelerate rapid and more accurate engineering.

It is interesting that there are many genes downregulated by the CRISPRi system but did not result in the isoprenol production enhancement. These genes might not be directly or indirectly associated with metabolic pathways (i.e., accumulation of precursors and cofactors to enhance isoprenol production and suppression of competing by-products). Nonetheless, it is important to note that genes which did not directly contribute to improving isoprenol production could also be the focus of future research. Further investigations would help to deepen our understanding of the metabolic pathways necessary for the production of desired bio-products.

## Data Availability

The data supporting the conclusions of this article will be made available by the authors, without undue reservation.
