# Supplementary material for: Enhancing isoprenol production by systematically tuning metabolic pathways using CRISPR interference in E. coli
Source: Front Bioeng Biotechnol. 2023 Nov 6;11:1296132. doi: 10.3389/fbioe.2023.1296132 (PMC10659101; doi:10.3389/fbioe.2023.1296132)
Supplement: Supplementary file 1 [file DataSheet1.docx]

*Supplementary Information*

**Enhancing isoprenol production by systematically tuning metabolic pathways using CRISPR interference in *E. coli***

Jinho Kim^1,2^, Taek Soon Lee^1,2, *^

^1^Joint BioEnergy Institute, 5885 Hollis Street, Emeryville, CA 94608, USA

^2^Biological Systems and Engineering Division, Lawrence Berkeley National Laboratory, Berkeley, CA 94720, USA

^*^Corresponding author: Dr. Taek Soon Lee, Joint BioEnergy Institute, 5885 Hollis St. 4^th^ floor, Emeryville, CA 94608, USA; Phone: +1-510-495-2470, Fax: +1-510-495-2629, E-mail: tslee@lbl.gov


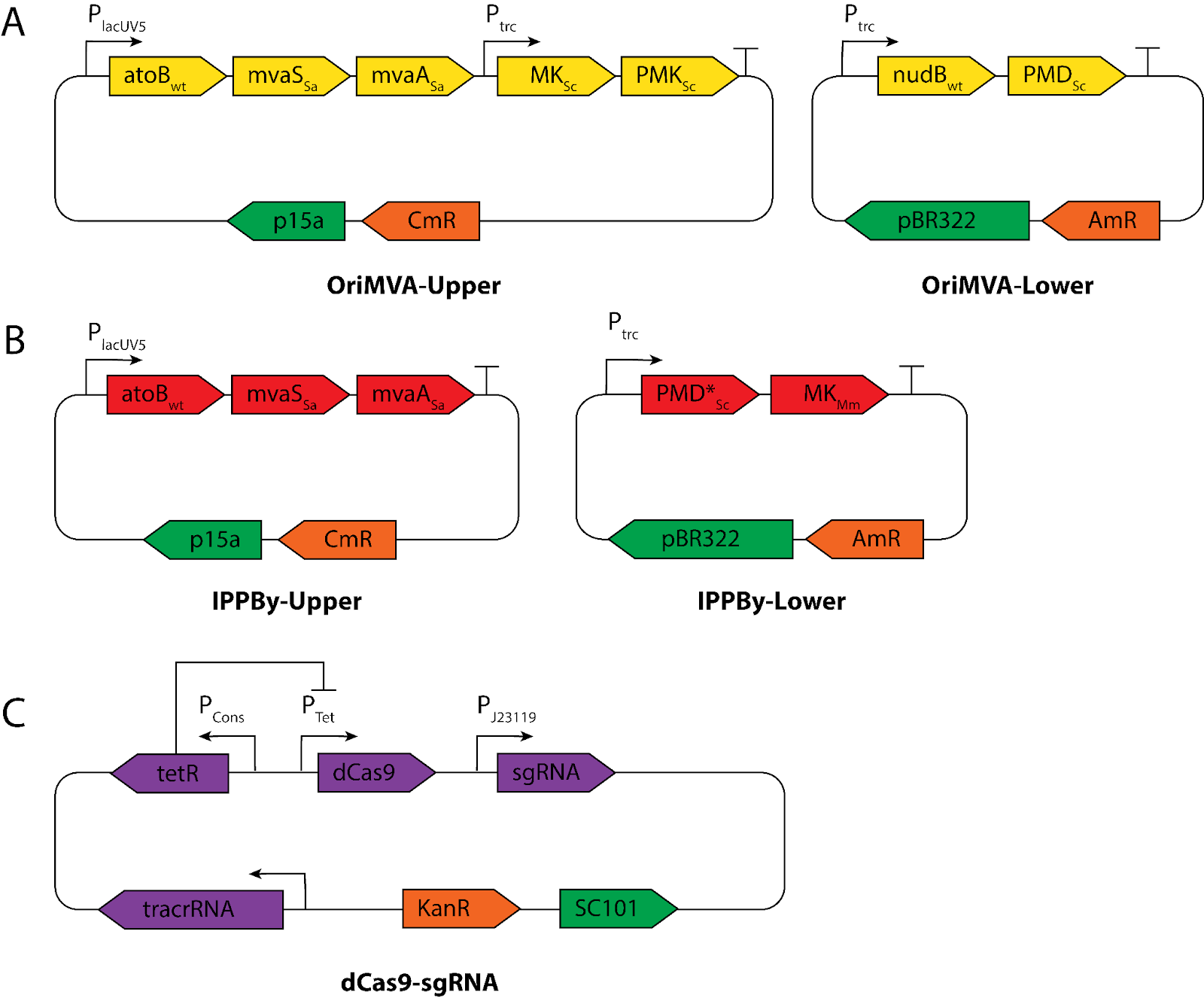


**Supplementary Figure S1.** Cartoons of the plasmids comprising A) the original MVA pathway, B) the IPP-bypass pathway, and C) the dCas9-sgRNA


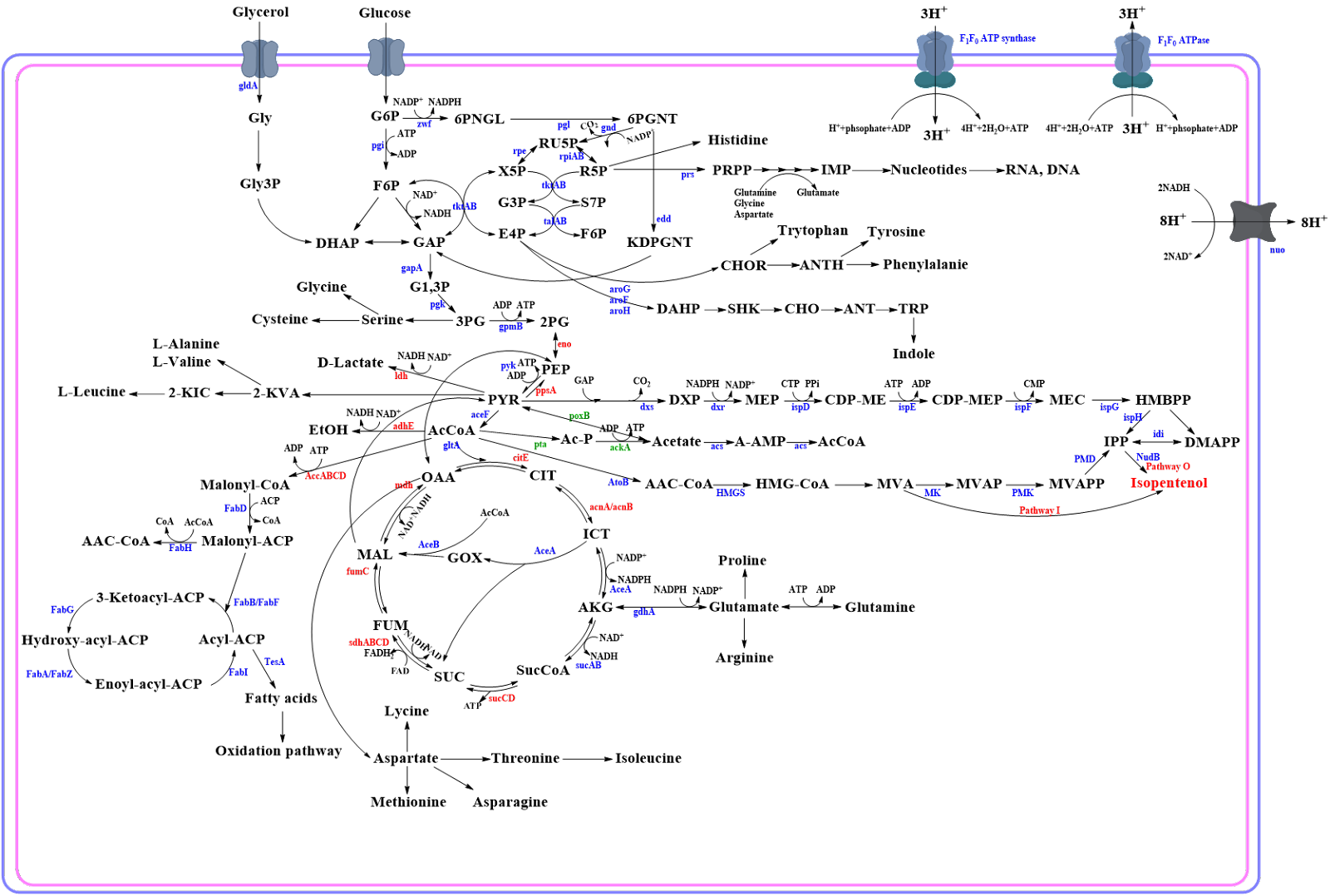


**Supplementary Figure S2.** Metabolic map of several pathways in *Escherichia coli*. The enzymes of interest including 32 gene knockdown targets of this work are noted in red and blue text.

**
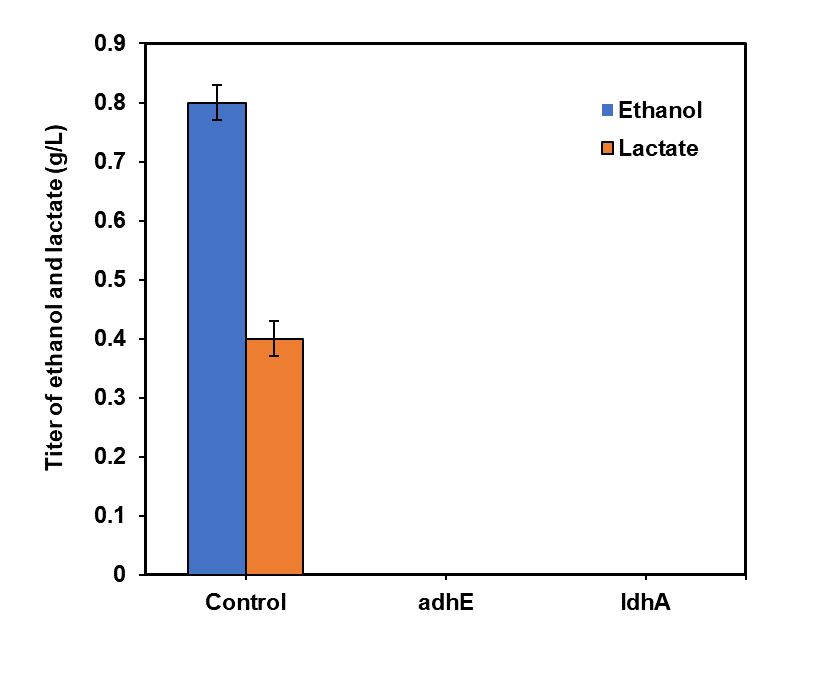
**

**Supplementary Figure S3.** Analysis of the ethanol and lactate. The ethanol and lactate production of strains harboring single gRNA, adhE and ldhA, individually along with the original MVA pathway grown in 5 mL M9-MOPS minimal medium supplemented with 20 g/L of glucose (n = 3).

**Supplementary Table S1.** Sequence of gRNAs used in this work

| **Target genes** | **Guide RNA sequence** | **Reference** |
| --- | --- | --- |
| *accA* | TCCAGCTCTGCAATCGGCTGTTCAAAATCA | [Tian et al., 2019](https://sciwheel.com/work/citation?ids=6985379&pre=&suf=&sa=0) |
| *ackA* | AGTTCAGAACCAGTACTAACTTACTCGACA | [Tian et al., 2019](https://sciwheel.com/work/citation?ids=6985379&pre=&suf=&sa=0) |
| *adhE* | GCTCTTGAGTGAAACTGGCATATTCACGCT | This study |
| *arcC* | TGCCCCCAAGAGCCACAACCAGTGTTTTCA | This study |
| *asnA* | AGCTAATTTGACGTTGTTTGGCAATGTAAG | [Tian et al., 2019](https://sciwheel.com/work/citation?ids=6985379&pre=&suf=&sa=0) |
| *citE* | GCGAAGCGGAAATCATTGGCAATCCTCCCA | This study |
| *dacA* | CCAGGCGCTTCATGATACGAGCGGAAAAAA | [Tian et al., 2019](https://sciwheel.com/work/citation?ids=6985379&pre=&suf=&sa=0) |
| *deoB* | CTTTGGCACAAGCTTCTGCGATATGACCCA | [Tian et al., 2019](https://sciwheel.com/work/citation?ids=6985379&pre=&suf=&sa=0) |
| *eutD* | GTCTAACGCATCCGGAAAAACCACTCTGGC | [Tian et al., 2019](https://sciwheel.com/work/citation?ids=6985379&pre=&suf=&sa=0) |
| *fabH* | CTTCGTATACATGTACGCTCAGTCACTTTT | This study |
| *gldA* | TGGATGTATTTACCCGGTGATTGAATAATG | [Tian et al., 2019](https://sciwheel.com/work/citation?ids=6985379&pre=&suf=&sa=0) |
| *ispA* | GGCCTGCTTAACGCAGGCTTCGAGTTGCTG | [Tian et al., 2019](https://sciwheel.com/work/citation?ids=6985379&pre=&suf=&sa=0) |
| *ldhA* | ACTTCTTGTCGTACTGTTTTGTGCTATAAA | This study |
| *mdh* | AGCGCCTGGCCAATACCGCCAGCAGCGCCG | [Tian et al., 2019](https://sciwheel.com/work/citation?ids=6985379&pre=&suf=&sa=0) |
| *menA* | GGTCGTAAACTTTCCAGCCACGCCTGAGTT | This study |
| *mqo* | CATTAAGCCCCACGGCCATCGAGAAGAGCA | [Tian et al., 2019](https://sciwheel.com/work/citation?ids=6985379&pre=&suf=&sa=0) |
| *pgl* | TCCAGACGTGAATTTGCTGGCTCTCAGGGC | This study |
| *poxB* | TCATGGTTCTCCATCTCCTGAATGTGATAA | [Tian et al., 2019](https://sciwheel.com/work/citation?ids=6985379&pre=&suf=&sa=0) |
| *ppc* | TGCCGAGCATACTGACATTACTACGCAATG | [Tian et al., 2019](https://sciwheel.com/work/citation?ids=6985379&pre=&suf=&sa=0) |
| *ppsA* | ACATCGAACAATCCTTTTGTGATAAATGAA | This study |
| *prpE* | ACGCCGGGCCTGCTCGGCCCAGAACTGCTC | [Tian et al., 2019](https://sciwheel.com/work/citation?ids=6985379&pre=&suf=&sa=0) |
| *pta* | TGGTTCCGGTAGGGATCAGCATAATAATAC | [Tian et al., 2019](https://sciwheel.com/work/citation?ids=6985379&pre=&suf=&sa=0) |
| *rssA* | TAGCACTATGTTAACCGAGCAGTAGCGATG | This study |
| *sdhABCD* | GAACCGGATGGTCTGTAGGTCCAGATTAAC | This study |
| *sucA* | TTAAGCATCTTTTTTATGCTTACTTCGCCG | This study |
| *sucB* | GGTGGCATCGGCTACGGATTCAGGCAGGTC | This study |
| *sucCD* | CGGAGTAGTACAGGCATAACCCACCGGTGC | This study |
| *thrC* | AAAACAGCCCCTGATTTTTGCCCAACCCCT | [Tian et al., 2019](https://sciwheel.com/work/citation?ids=6985379&pre=&suf=&sa=0) |
| *ubiA* | CCGTTGTACTAAGAGGAAAAAAATATGGAG | This study |
| *yahl* | GCTCTTTCATGTTGTTATGTCCGCTACAGA | This study |
| *yhfW* | GTGACCACATGTATTCGCTCCCGCATCTTG | This study |

**Supplementary Table S2.** Strains used in this study

| **Strain Name** | **Strain Number from JBEI Registry** | **Description (*E. coli* gene names are the target gRNA sequences from Supplementary Table S1)** | **Reference** |
| --- | --- | --- | --- |
| **Original Mevalonate pathway strains for isoprenol biosynthesis** | | | |
| accA | JPUB_011331 | pTet-dCas9-pJ23119-accA, pSC101, KanR | [(Tian et al., 2019)](https://sciwheel.com/work/citation?ids=6985379&pre=&suf=&sa=0) |
| ackA | JPUB_011309 | pTet-dCas9-pJ23119-ackA, pSC101, KanR | [(Tian et al., 2019)](https://sciwheel.com/work/citation?ids=6985379&pre=&suf=&sa=0) |
| adhE | JBEI_239617 | pTet-dCas9-pJ23119-adhE, pSC101, KanR | This study |
| arcC | JPUB_011323 | pTet-dCas9-pJ23119-arcC, pSC101, KanR | [(Tian et al., 2019)](https://sciwheel.com/work/citation?ids=6985379&pre=&suf=&sa=0) |
| asnA | JPUB_011347 | pTet-dCas9-pJ23119-asnA, pSC101, KanR | [(Tian et al., 2019)](https://sciwheel.com/work/citation?ids=6985379&pre=&suf=&sa=0) |
| citE | JBEI_239624 | pTet-dCas9-pJ23119-citE, pSC101, KanR | This study |
| dacA | JPUB_011357 | pTet-dCas9-pJ23119-dacA, pSC101, KanR | [(Tian et al., 2019)](https://sciwheel.com/work/citation?ids=6985379&pre=&suf=&sa=0) |
| deoB | JPUB_011335 | pTet-dCas9-pJ23119-deoB, pSC101, KanR | [(Tian et al., 2019)](https://sciwheel.com/work/citation?ids=6985379&pre=&suf=&sa=0) |
| eutD | JPUB_011333 | pTet-dCas9-pJ23119-eutD, pSC101, KanR | [(Tian et al., 2019)](https://sciwheel.com/work/citation?ids=6985379&pre=&suf=&sa=0) |
| fabH | JBEI_239618 | pTet-dCas9-pJ23119-fabH, pSC101, KanR | This study |
| gldA | JPUB_011351 | pTet-dCas9-pJ23119-gldA, pSC101, KanR | [(Tian et al., 2019)](https://sciwheel.com/work/citation?ids=6985379&pre=&suf=&sa=0) |
| ispA | JPUB_011343 | pTet-dCas9-pJ23119-ispA, pSC101, KanR | [(Tian et al., 2019)](https://sciwheel.com/work/citation?ids=6985379&pre=&suf=&sa=0) |
| ldhA | JBEI_239619 | pTet-dCas9-pJ23119-ldhA, pSC101, KanR | This study |
| mdh | JPUB_011345 | pTet-dCas9-pJ23119-mdh, pSC101, KanR | [(Tian et al., 2019)](https://sciwheel.com/work/citation?ids=6985379&pre=&suf=&sa=0) |
| menA | JBEI_239621 | pTet-dCas9-pJ23119-menA, pSC101, KanR | This study |
| mqo | JPUB_011353 | pTet-dCas9-pJ23119-mqo, pSC101, KanR | [(Tian et al., 2019)](https://sciwheel.com/work/citation?ids=6985379&pre=&suf=&sa=0) |
| pgl | JBEI_239625 | pTet-dCas9-pJ23119-pgl, pSC101, KanR | This study |
| poxB | JPUB_013307 | pTet-dCas9-pJ23119-poxB, pSC101, KanR | This study |
| ppc | JPUB_011337 | pTet-dCas9-pJ23119-ppc, pSC101, KanR | [(Tian et al., 2019)](https://sciwheel.com/work/citation?ids=6985379&pre=&suf=&sa=0) |
| ppsA | JBEI_239615 | pTet-dCas9-pJ23119-ppsA, pSC101, KanR | This study |
| prpE | JPUB_011349 | pTet-dCas9-pJ23119-prpE, pSC101, KanR | [(Tian et al., 2019)](https://sciwheel.com/work/citation?ids=6985379&pre=&suf=&sa=0) |
| pta | JPUB_011311 | pTet-dCas9-pJ23119-pta, pSC101, KanR | [(Tian et al., 2019)](https://sciwheel.com/work/citation?ids=6985379&pre=&suf=&sa=0) |
| rssA | JBEI_239584 | pTet-dCas9-pJ23119-rssA, pSC101, KanR | This study |
| sdhABCD | JBEI_239627 | pTet-dCas9-pJ23119-sdhABCD, pSC101, KanR | This study |
| sucA | JBEI_239626 | pTet-dCas9-pJ23119-sucA, pSC101, KanR | This study |
| sucB | JBEI_239622 | pTet-dCas9-pJ23119-sucB, pSC101, KanR | This study |
| sucCD | JBEI_239623 | pTet-dCas9-pJ23119-sucCD, pSC101, KanR | This study |
| thrC | JPUB_011341 | pTet-dCas9-pJ23119-thrC, pSC101, KanR | [(Tian et al., 2019)](https://sciwheel.com/work/citation?ids=6985379&pre=&suf=&sa=0) |
| ubiA | JBEI_252597 | pTet-dCas9-pJ23119-ubiA, pSC101, KanR | This study |
| yahl | JPUB_011325 | pTet-dCas9-pJ23119-yahl, pSC101, KanR | [(Tian et al., 2019)](https://sciwheel.com/work/citation?ids=6985379&pre=&suf=&sa=0) |
| yhfW | JPUB_011339 | pTet-dCas9-pJ23119-yhfW, pSC101, KanR | [(Tian et al., 2019)](https://sciwheel.com/work/citation?ids=6985379&pre=&suf=&sa=0) |
| yqeA | JPUB_011327 | pTet-dCas9-pJ23119-yqeA, pSC101, KanR | [(Tian et al., 2019)](https://sciwheel.com/work/citation?ids=6985379&pre=&suf=&sa=0) |
| adhE-fabH | JBEI_238271 | pTet-dCas9-pJ23119-adhE-fabH, pSC101, KanR | This study |
| adhE-menA | JBEI_238267 | pTet-dCas9-pJ23119-adhE-menA, pSC101, KanR | This study |
| adhE-ubiA | JBEI_238296 | pTet-dCas9-pJ23119-adhE-ubiA, pSC101, KanR | This study |
| fabH-menA | JBEI_252602 | pTet-dCas9-pJ23119-fabH-menA, pSC101, KanR | This study |
| fabH-ubiA | JBEI_252600 | pTet-dCas9-pJ23119-fabH-ubiA, pSC101, KanR | This study |
| ldhA-adhE | JBEI_238273 | pTet-dCas9-pJ23119-ldhA-adhE, pSC101, KanR | This study |
| ldhA-fabH | JBEI_238272 | pTet-dCas9-pJ23119-ldhA-fabH, pSC101, KanR | This study |
| ldhA-menA | JBEI_238296 | pTet-dCas9-pJ23119-ldhA-menA, pSC101, KanR | This study |
| ldhA-ubiA | JBEI_238293 | pTet-dCas9-pJ23119-ldhA-ubiA, pSC101, KanR | This study |
| menA-ubiA | JBEI_238300 | pTet-dCas9-pJ23119-menA-ubiA, pSC101, KanR | This study |
| adhE-fabH-ldhA | JBEI_252614 | pTet-dCas9-pJ23119-adhE-fabH-ldhA, pSC101, KanR | This study |
| adhE-fabH-menA | JBEI_252612 | pTet-dCas9-pJ23119-adhE-fabH-ldhA, pSC101, KanR | This study |
| menA-ubiA-fabH | JBEI_252613 | pTet-dCas9-pJ23119-menA-ubiA-fabH, pSC101, KanR | This study |
| **IPP-bypass pathway strains** | | | |
| accA | JPUB_011331 | pTet-dCas9-pJ23119-accA, pSC101, KanR | This study |
| ackA | JPUB_011309 | pTet-dCas9-pJ23119-ackA, pSC101, KanR | This study |
| adhE | JBEI_239546 | pTet-dCas9-pJ23119-adhE, pSC101, KanR | This study |
| arcC | JPUB_011323 | pTet-dCas9-pJ23119-arcC, pSC101, KanR | This study |
| asnA | JPUB_011347 | pTet-dCas9-pJ23119-asnA, pSC101, KanR | This study |
| citE | JBEI_239545 | pTet-dCas9-pJ23119-citE, pSC101, KanR | This study |
| dacA | JPUB_011357 | pTet-dCas9-pJ23119-dacA, pSC101, KanR | This study |
| deoB | JPUB_011335 | pTet-dCas9-pJ23119-deoB, pSC101, KanR | This study |
| eutD | JPUB_011333 | pTet-dCas9-pJ23119-eutD, pSC101, KanR | This study |
| fabH | JBEI_239585 | pTet-dCas9-pJ23119-fabH, pSC101, KanR | This study |
| gldA | JPUB_011351 | pTet-dCas9-pJ23119-gldA, pSC101, KanR | This study |
| ispA | JPUB_011343 | pTet-dCas9-pJ23119-ispA, pSC101, KanR | This study |
| ldhA | JBEI_239586 | pTet-dCas9-pJ23119-ldhA, pSC101, KanR | This study |
| mdh | JPUB_011345 | pTet-dCas9-pJ23119-mdh, pSC101, KanR | This study |
| menA | JBEI_239582 | pTet-dCas9-pJ23119-menA, pSC101, KanR | This study |
| mqo | JPUB_011353 | pTet-dCas9-pJ23119-mqo, pSC101, KanR | This study |
| pgl | JBEI_239572 | pTet-dCas9-pJ23119-pgl, pSC101, KanR | This study |
| poxB | JPUB_013307 | pTet-dCas9-pJ23119-poxB, pSC101, KanR | This study |
| ppc | JPUB_011337 | pTet-dCas9-pJ23119-ppc, pSC101, KanR | This study |
| ppsA | JBEI_239544 | pTet-dCas9-pJ23119-ppsA, pSC101, KanR | This study |
| prpE | JPUB_011349 | pTet-dCas9-pJ23119-prpE, pSC101, KanR | This study |
| pta | JPUB_011311 | pTet-dCas9-pJ23119-pta, pSC101, KanR | This study |
| rssA | JBEI_239578 | pTet-dCas9-pJ23119-rssA, pSC101, KanR | This study |
| sdhABCD | JBEI_239630 | pTet-dCas9-pJ23119-sdhABCD, pSC101, KanR | This study |
| sucA | JBEI_252598 | pTet-dCas9-pJ23119-sucA, pSC101, KanR | This study |
| sucB | JBEI_239628 | pTet-dCas9-pJ23119-sucB, pSC101, KanR | This study |
| sucCD | JBEI_252599 | pTet-dCas9-pJ23119-sucCD, pSC101, KanR | This study |
| thrC | JPUB_011341 | pTet-dCas9-pJ23119-thrC, pSC101, KanR | This study |
| ubiA | JBEI_239581 | pTet-dCas9-pJ23119-ubiA, pSC101, KanR | This study |
| yahl | JPUB_011325 | pTet-dCas9-pJ23119-yahl, pSC101, KanR | This study |
| yhfW | JPUB_011339 | pTet-dCas9-pJ23119-yhfW, pSC101, KanR | This study |
| yqeA | JPUB_011327 | pTet-dCas9-pJ23119-yqeA, pSC101, KanR | This study |
| adhE-fabH | JBEI_252606 | pTet-dCas9-pJ23119-adhE-fabH, pSC101, KanR | This study |
| adhE-menA | JBEI_238314 | pTet-dCas9-pJ23119-adhE-menA, pSC101, KanR | This study |
| adhE-ubiA | JBEI_238319 | pTet-dCas9-pJ23119-adhE-ubiA, pSC101, KanR | This study |
| fabH-menA | JBEI_238322 | pTet-dCas9-pJ23119-fabH-menA, pSC101, KanR | This study |
| fabH-ubiA | JBEI_252601 | pTet-dCas9-pJ23119-fabH-ubiA, pSC101, KanR | This study |
| ldhA-adhE | JBEI_238321 | pTet-dCas9-pJ23119-ldhA-adhE, pSC101, KanR | This study |
| ldhA-fabH | JBEI_252605 | pTet-dCas9-pJ23119-ldhA-fabH, pSC101, KanR | This study |
| ldhA-menA | JBEI_252604 | pTet-dCas9-pJ23119-ldhA-menA, pSC101, KanR | This study |
| ldhA-ubiA | JBEI_252603 | pTet-dCas9-pJ23119-ldhA-ubiA, pSC101, KanR | This study |
| menA-ubiA | JBEI_238318 | pTet-dCas9-pJ23119-menA-ubiA, pSC101, KanR | This study |
| ppsA-adhE | JBEI_238340 | pTet-dCas9-pJ23119-ppsA-adhE, pSC101, KanR | This study |
| ppsA-fabH | JBEI_238317 | pTet-dCas9-pJ23119-ppsA-fabH, pSC101, KanR | This study |
| ppsA-ldhA | JBEI_238304 | pTet-dCas9-pJ23119-ppsA-ldh5A, pSC101, KanR | This study |
| ppsA-menA | JBEI_238310 | pTet-dCas9-pJ23119-ppsA-menA, pSC101, KanR | This study |
| ppsA-ubiA | JBEI_238305 | pTet-dCas9-pJ23119-ppsA-ubiA, pSC101, KanR | This study |
| adhE-fabH-ldhA | JBEI_252615 | pTet-dCas9-pJ23119-adhE-fabH-ldhA, pSC101, KanR | This study |
| adhE-fabH-menA | JBEI_252616 | pTet-dCas9-pJ23119-adhE-fabH-menA, pSC101, KanR | This study |
| adhE-fabH-ubiA | JBEI_252618 | pTet-dCas9-pJ23119-adhE-fabH-ubiA, pSC101, KanR | This study |
